# Glycan Signatures on Neutrophils in an Equine Model for Autoimmune Uveitis

**DOI:** 10.3390/biom15101444

**Published:** 2025-10-12

**Authors:** Carolin J. Sprenzel, Barbara Amann, Cornelia A. Deeg, Roxane L. Degroote

**Affiliations:** Chair of Physiology, Department of Veterinary Sciences, Ludwig-Maximilians-University Munich, D-82152 Martinsried, Germany

**Keywords:** O-glycosylation, surface glycome, Jacalin, neutrophils, equine recurrent uveitis, ERU, autoimmune uveitis

## Abstract

Glycosylation of surface proteins is a crucial post-translational modification that reflects the activation status of neutrophils, the predominant leukocyte subset in humans and horses. Neutrophils have emerged as active contributors to diseases mediated by the adaptive immune system, such as equine recurrent uveitis (ERU), a sight-threatening disease in horses and a unique model for studying the pathogenesis of autoimmune uveitis in humans. Since changes in surface glycosylation can impact neutrophil function, we were interested in the surface glycosylation landscape on neutrophils from healthy horses and the potential changes in surface glyco-signatures in ERU. Using 35 different plant lectins, we outlined a profile of surface-exposed glycan moieties on equine neutrophils and detected significantly increased O-glycosylation in a diseased state through Jacalin (JAC) binding via flow cytometry. Subsequent molecular weight comparison of JAC pull-down assay data and neutrophil proteomics indicated the surface proteins Integrin beta-2 and CUB domain-containing protein 1 as potential anchors for increased O-glycan levels in ERU. These findings give novel insights into neutrophil surface glycosylation in health and disease and propose O-glycosylation as a possible biomarker for autoimmune uveitis.

## 1. Introduction

The cell surface is characterized by membrane proteins, which fulfill various functions like transport, signaling, cell–cell-communication, enzymatic activity or identification of cell subsets via glycoproteins. For example, human blood types can be determined by glycoproteins on the cell surface of erythrocytes [1]. After transcription, translation, and folding, membrane proteins usually undergo post-translational modifications (PTM). One of the most important PTMs of membrane proteins is glycosylation [2].

Glycosylation describes the attachment of glycans to proteins. The two major types of glycosylation are N- and O-glycosylation [3,4]. While N-glycosylation is characterized by glycan-attachment to the nitrogen atom of asparagine residues, O-glycosylation describes the attachment of glycans to the oxygen atom of serine or threonine residues [5]. The role of cell surface glycosylation in autoimmune diseases is an emerging area of interest. In patients suffering from systemic lupus erythematosus, alterations in numerous glycans were discovered in different immune cell subsets. These alterations were described to correlate with the severity of systemic lupus erythematosus, indicating a connection between surface glycome composition and disease pathogenesis in humans [6]. Aberrant surface glycosylation may even serve as a biomarker for autoimmune diseases like multiple sclerosis in humans [7]. Furthermore, PTMs might be involved in autoimmune uveitis (AU) pathogenesis. For example, Yin Yang 1 lactylation, a PTM describing the addition of a lactate molecule to the protein Yin Yang 1, was described to enhance microglial functions in the retina of a murine experimental autoimmune uveitis model by upregulation of inflammatory genes [8].

AU is characterized by chronic recurrent inflammation of the eye, caused by invading immune cells from the peripheral bloodstream, which overcome the blood–retinal barrier and cause severe destruction of inner eye structures [9]. Treatment options are limited to glucocorticoids and immunosuppressants, which can cause detrimental side effects in patients [10]. Equine recurrent uveitis (ERU) resembles AU in many aspects of pathophysiology and serves as a valuable spontaneous model [11,12,13]. Unlike AU, treatment of horses affected by ERU is performed only during active uveitis [13,14,15]. Therefore, the stages of quiescence allow investigation of untreated immune cells in ERU. Although ERU and AU are described as mainly T-cell driven diseases [16,17], the presences of neutrophils in eyes from induced mouse and rat models for AU [18,19,20] and in ocular tissue from ERU horses [21] indicates a possible role of these cells in disease pathogenesis, especially since these cells show a pre-activated phenotype even in the quiescent stage of disease [22].

Neutrophils are the most abundant leukocyte subset in human and equine blood. As the first line of defense against pathogens, these cells play a crucial role in inflammation [23] by performing their well-described repertoire of defense mechanisms: phagocytosis, degranulation, production of reactive oxygen species or neutrophil extracellular traps [24,25]. Excessive neutrophil effector functions can lead to hyperinflammation, resulting in tissue damage. This explains why an adequate regulation of neutrophil immune response is essential for health [26].

Since PTMs such as glycosylation can modulate immune cell function, we aimed to unravel the surface glycome of equine neutrophils and investigate potential changes in ERU, which may provide insights into novel activation mechanisms of neutrophils in autoimmune uveitis. Aberrant glycan moieties on neutrophils from horses with ERU may be suitable to serve as biomarkers for disease prediction and monitoring or even as targets for therapeutic intervention.

## 2. Materials and Methods

### 2.1. Animals and Cell Preparation

Blood was drawn from 13 control horses and 22 equine recurrent uveitis (ERU) cases for all experiments within this study. Some of the horses in both groups were used in multiple assays. ERU was diagnosed by experienced clinicians from the Equine Clinic at LMU Munich according to typical clinical signs of recurrent uveitis in addition to a documentation of at least two recent episodes of inflammation in the affected eye [27]. Horses with end-stage recurrent uveitis (blindness, enucleation) were excluded from the study. During blood withdrawal, all ERU cases were in a quiescent stage of disease to undergo a therapeutic procedure (pars plana vitrectomy). Horses were classified as ophthalmologically healthy when no history of ocular inflammation was reported by the owner, and a standard clinical examination performed by a veterinarian revealed no evidence of pathophysiological alterations within the eye. Collection of blood was permitted by the local authority, Regierung von Oberbayern (Permit number: ROB-55.2-2532.Vet_03-22-37).

All equine whole blood samples were taken from the vena jugularis and stored in tubes containing heparin sodium (50 I.U. per ml blood; Ratiopharm, Ulm, Germany). Following initial sedimentation of erythrocytes at room temperature, the leukocyte-enriched plasma was carefully layered onto Pancoll separation solution (PanBiotech, Aidenbach, Germany), and leukocyte subsets were separated using density gradient centrifugation (room temperature (RT), 350× *g*, 25 min, brake off). The neutrophils were extracted from the sediment at the bottom of the density gradient. After removing the remaining layers of plasma and pancoll, the sediment was carefully resuspended in phosphate-buffered saline (PBS; NaCl 136.9 mM (Sigma-Aldrich, Taufkirchen, Germany), Na_2_HPO_4_ × 2H_2_O 8.1 mM (AppliChem, Darmstadt, Germany), KH_2_PO_4_ 1.4 mM (Sigma-Aldrich, Taufkirchen, Germany), KCl 2.6 mM (Sigma-Aldrich, Taufkirchen, Germany); pH 7.4). The remaining erythrocytes were lysed twice with 20 mL 0.2% sodium chloride (NaCl, Sigma-Aldrich, Taufkirchen, Germany) for 30 s, followed by 20 mL 1.6% NaCl to recover isotonicity and a washing step (RT, 400× *g*, 10 min). The resulting neutrophils were resuspended in PBS. Cell counts for neutrophils were determined in a Neubauer improved counting chamber using trypan blue solution (VWR Life Science, Darmstadt, Germany) for exclusion of dead cells.

### 2.2. Analysis of Surface Glycosylation by Flow Cytometry

Neutrophils from seven controls and eleven ERU cases were seeded into a 96-well U-bottom plate with a cell count of 5 × $10^{5}$ per well after excluding dead cells. All lectins were incubated for 15 min at RT and subsequently washed with PBS (RT, 400× *g*, 2 min). To exclude dead cells, Viobility 400/452 Fixable Dye (Miltenyi Biotec, Bergisch Gladbach, Germany) was used. Cells were stained with 35 biotinylated plant lectins (1 µL/mL) to assess glycan-binding profiles (lectins used are from Biozol, Eching, Germany, or Linaris, Dossenheim, Germany): Aleuria aurantia lectin (AAL), Agaricus bisporus lectin (ABL), Amaranthus caudatus lectin (ACL), Banana lectin (BanLec), Bauhinia purpurea lectin (BPL), Concanavalin A (ConA), Dolichos biflorus agglutinin (DBA), Datura stramonium lectin (DSL), Erythrina cristagalli lectin (ECL), Galanthus nivalis lectin (GNL), Griffonia simplicifolia lectin I (GSL-I), Griffonia simplicifolia lectin I Isolectin B4 (GSL-I-B4), Griffonia simplicifolia lectin II (GSL-II), Hippeastrum hybrid lectin (HHL), Jacalin (JAC), Lens culinaris agglutinin (LCA), Lycopersicon esculentum (tomato) lectin (LEL), Lotus tetragonolobus lectin (LTL), Maackia amurensis lectin II (MAL-II), Narcissus pseudonarcissus lectin (NPL), Phaseolus vulgaris erythroagglutinin (PHA-E), Phaseolus vulgaris leucoagglutinin (PHA-L), Peanut agglutinin (PNA), Pisum sativum agglutinin (PSA), Psophocarpus tetragonolobus lectin (PTL), Ricinus communis agglutinin I (RCA-I), Soybean agglutinin (SBA), Sophora japonica agglutinin (SJA), Sambuccus nigra agglutinin (SNA), Solanum tuberosum (potato) lectin (STL), succinylated Wheat germ agglutinin (sWGA), Ulex europaeus agglutinin I (UEA-I), Vicia villosa lectin (VVA), Wisteria floribunda lectin (WFL), Wheat germ agglutinin (WGA). More detailed information about the plant lectins is displayed in Table 1. Lectin binding was visualized using Streptavidin iFluor 488 and Streptavidin iFluor 555 (Biomol, Hamburg, Germany, 1:200). Cells were subsequently fixed with 1% paraformaldehyde (PFA; Sigma-Aldrich, Darmstadt, Germany).

To assess the glycosylation pattern of the neutrophil cell surface, measurements were performed using a NovoCyte Quanteon 4016 flow cytometer (Agilent Technologies, Waldbronn, Germany). Data were analyzed with Flowlogic Software V7 (Inivai Technologies, Mentone Victoria, Australia) and NovoExpress Software version 1.5.0 (Agilent Technologies). Lectin binding expression intensities were quantified using mean fluorescence intensity (MFI) values and normalized to respective unstained cells per animal.

### 2.3. Immunocytochemistry

Neutrophils (5 × 10^5^ cells per well) from three controls and three ERU cases were seeded into a 96-well U-bottom plate and incubated with the biotinylated plant lectins DBA, BanLec and JAC (Biozol, Eching, Germany, 1 µg/mL each) for 15 min at RT. Neutrophils were then washed (RT, 400× *g*, 2 min). To visualize plant lectin binding on the cell surface, Streptavidin iFluor488 (Biomol, Hamburg, Germany, 1:200) was added and incubated for 15 min at RT, followed by a final washing step (RT, 400× *g*, 2 min). Unspecific binding of Streptavidin iFluor488 was assessed via single staining without lectins. Cell nuclei were stained with 4′, 6-Diamidin-2-phenylindol (DAPI; Invitrogen, Karlsruhe, Germany; 1:1000) and fixed in PBS containing 1% PFA. Half of the cell suspension (2.5 × 10^5^ cells) was transferred to microscope slides (RT, 300× *g*, 10 min) and embedded in fluoromount medium (Serva, Heidelberg; Germany), adding coverslips. Visualization was achieved with a Leica DMi8 microscope (Leica Microsystems, Wetzlar, Germany). Background removal was performed via THUNDER Computational Clearing (Leica Microsystems, Wetzlar, Germany) (Supplementary Materials: RawData ICC). Fluorescent images were quantified with ImageJ (Version 1.54g) [28]. For ImageJ analysis, signals from cell nucleus staining were excluded and fluorescent images of the lectin signals were converted into a greyscale format. After background subtraction, the pixel intensity per lectin signal on each cell was measured as mean gray value. A total of 10 cells per lectin were used for quantification.

For evaluation of O-glycan prediction from proteome data, neutrophils from one control and two ERU cases were used. Staining and visualization was performed as described (see above) with the following antibodies: anti-CD18 (Integrin beta-2; Clone CLB-LFA-1/1; Thermo Fisher Scientific, Ulm, Germany; 1:50), goat anti-mouse IgG Alexa568 (Invitrogen, Karlsruhe, Germany; 1:500), biotinylated plant lectin JAC (Biozol, Eching, Germany; 1 µg/mL), Streptavidin Alexa488 (Invitrogen, Karlsruhe, Germany; 1:500) (Supplementary Materials: RawData ICC).

### 2.4. JAC Pull-Down

Neutrophils from three controls and three ERU cases were lysed in CHAPS-lysis buffer (2% CHAPS (Carl Roth, Karlsruhe, Germany) in Tris-buffered saline with 1× Roche complete protease inhibitor (Sigma-Aldrich, Taufkirchen, Germany)) using 500 µL per 5 × 10^5^ cells. CHAPS-lysis buffer was incubated for 30 min on ice and mixed repeatedly. Lysed cells were loaded twice onto a QIAshredder homogenizer (Quiagen, Stockach, Germany) and centrifuged (23 °C, 16,100 × *g*, 2 min). Protein concentration was determined by Bicinchonin Acid-Assay (Thermo Fisher Scientific, Ulm, Germany). Agarose bound JAC (Biozol, Eching, Germany) was transferred to low-binding cups (Sarstedt, Nümbrecht, Germany) and washed (RT, 1800× *g*, 30 s) five times with 250 µL TRIS-buffer (175 mM TRIS (Carl Roth, Karlsruhe, Germany), pH 7.5). Agarose beads were mixed with 1 mg neutrophil lysate, and the mixture was rotated overnight at 4 °C. The next day, samples were centrifuged (RT, 1000× *g*, 2 min) and the supernatants were transferred to new low-binding cups. The precipitates were washed three times with PBS (RT, 1000× *g*, 1 min) before adding Laemmli buffer (without dithiothreitol and glycerin: 50 mM Tris-HCl, 1% SDS (both AppliChem, Darmstadt, Germany), 10% glycerol (SERVA, Heidelberg, Germany), 4 mM 2-mercaptoethanol, bromophenol blue (both Sigma-Aldrich, Taufkirchen, Germany)) and heating the samples to 95 °C for 10 min while shaking (400× *g*). Samples were centrifuged (RT, 1000× *g*, 2 min) and the eluates were collected.

Proteins were separated by 1D SDS-PAGE on 10% gels with either 20 µL of eluate, 2 µL of supernatant, or 5 µg of lysate per slot. Serva Triple Color Protein Standard III (Serva Electrophoresis, Heidelberg, Germany) was used as a molecular weight marker. After electrophoresis, gels were briefly washed in Aqua bidest and proteins were detected overnight by a modified Coomassie Brilliant Blue G250 (Fluka, Buchs, Switzerland)-based staining method [29]. Visualization of stained SDS gels was performed with Amersham Imager680, Analysis2.0 (GE Healthcare, Freiburg, Germany) (Supplementary Materials: RawData WB). The exact molecular weight of protein bands of interest was determined by generating a graph based on the migration distance of the protein marker bands (distance protein marker band migration divided by total migration distance on gel) and subsequently allocating the migration distance of protein bands of interest on the graph (Figure S1).

Quantification was performed with ImageJ (Version 1.54g) [28]. Prior to ImageJ analysis, the scanned gel images were converted into a grayscale format. Pixel intensity per band was measured after background subtraction.

### 2.5. JAC Inhibition

Neutrophils from seven horses were seeded into a 96-well U-bottom plate with a cell count of 5 × 10^5^ per well. To inhibit JAC binding to neutrophil surface glycans, FITC-conjugated JAC (Biozol, Eching, Germany) was incubated with the saccharides galactose, mannose, and sucrose (Merck KGaA, Darmstadt, Germany), diluted to a concentration of 20% in PBS for 30 min. Neutrophils were then stained with pre-treated JAC (1 µL/mL) for 15 min at RT and subsequently washed with PBS (RT, 400× *g*, 2 min).

Measurements were performed using a NovoCyte Quanteon 4016 flow cytometer (Agilent Technologies, Waldbronn, Germany). Data were analyzed with Flowlogic Software V7 (Inivai Technologies, Mentone Victoria, Australia) and NovoExpress Software version 1.5.0 (Agilent Technologies). JAC binding intensities (MFI) of saccharide-pre-treated JAC were normalized to binding intensities of untreated JAC.

### 2.6. Prediction of O-GalNAc Glycosylation and Evaluation of Neutrophil Proteome Data

Mass spectrometry analysis, protein identification and label-free quantification were conducted using a dataset previously generated and published by our group [22]. For the identification of candidate proteins, we reanalyzed this published dataset (raw data have been deposited to the ProteomeXchange Consortium via the PRIDE repository with the dataset identifier: PXD013648 [30]). This approach ensured consistency and reproducibility based on our established workflows. Prediction of O-GalNAc glycosylation was performed using NetOGlyc 4.0 [31]. Proteins were classified as O-glycosylated if they contained multiple predicted glycosylation sites with a probability score ≥0.5.

### 2.7. Statistical Analysis

To assess Gaussian distribution of the data, the Kolmogorov–Smirnov (KS) test was applied. For data showing normal distribution (KS *p* > 0.05), statistical differences between two groups were evaluated using Student’s t-test. Equality of variances was tested using the F-test; in cases of significantly different variances or unequal group sizes, Welch’s correction was applied. For data not following a normal distribution, the non-parametric Mann–Whitney U test was used. Results were considered statistically significant at *p* ≤ 0.05. Statistical significance was indicated as follows: * *p* ≤ 0.05, ** *p* ≤ 0.01 and *** *p* ≤ 0.001, **** *p* ≤ 0.0001. All statistical analyses and graphical representations were performed using GraphPad Prism software (version 5.04; GraphPad Software, San Diego, CA, USA). Outliers were identified using Grubbs’ test and excluded from further analysis if flagged as significant.

## 3. Results

### 3.1. Defining the Surface Glycome of Equine Neutrophils

**Figure 1:**
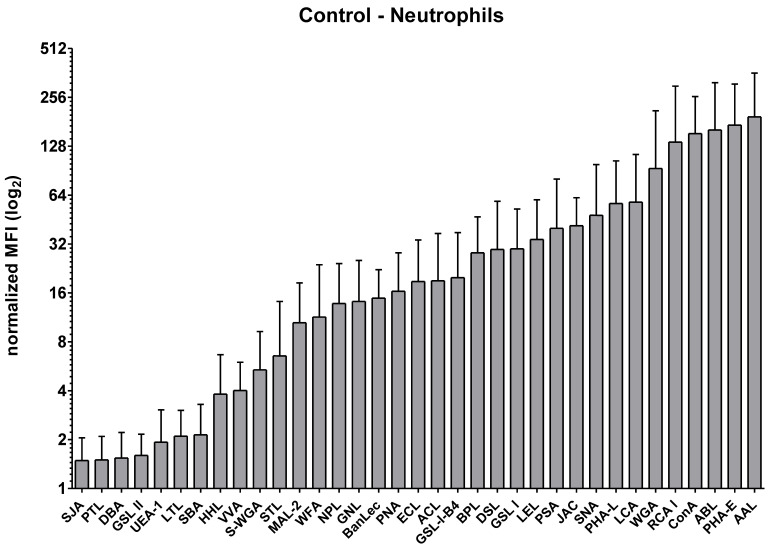
Surface glycosylation landscape of neutrophils from healthy control horses (*n* = 7; except AAL, ABL, ACL, GSL II, STL, WGA: *n* = 6 and DSL, JAC, LCA, LEL, PSA, RCA, SNA: *n* = 5; data was obtained in six independent experiments). Binding intensity of all 35 biotinylated plant lectins used in flow cytometry assays is shown with bar graphs. Mean fluorescence intensity with standard deviation (MFI ± SD) of lectin binding was normalized to respective unstained cells per animal. The *y*-axis is displayed on a log_2_ scale to account for differences in binding intensity across several orders of magnitude. Lectins are arranged from low binding to high binding intensity along the *x*-axis.

To assess the surface glycosylation of neutrophils from control horses, a panel of 35 plant lectins was employed. These lectins specifically bind to well-defined glycan structures, enabling a detailed profiling of surface-exposed carbohydrate moieties. The screening revealed a heterogeneous glycosylation pattern: while certain glycan structures exhibited high surface abundance, others were barely detectable or entirely absent. Notably, very low binding was observed for lectins recognizing terminal poly- or multiantennary type 2 LacNAc or LacdiNAc (SJA, VVA), carbohydrate structures containing (α-1,3) linked Gal and (α-1,2) linked fucose (PTL), terminal GalNAc-α1,3-GalNAc (DBA), oligosaccharides with terminal α- or β-linked GalNAc (SBA, VVA), α- or β-linked GlcNAc (GSL II), (α-1,2) linked fucose to (β-1,4) linked galactose (UEA-1), (α-1,3) linked fucose containing oligosaccharides (LTL), and terminal α-mannose structures (HHL) (Figure 1, Table 1 and Table S1). This absence of binding may indicate either a true lack of these glycan structures on the neutrophil surface or a spatial conformation that masks lectin accessibility.

In contrast to the absent glycan structures, several carbohydrate motifs were accessible on the neutrophil surface and yielded strong lectin-binding signals. These included β-galactose, terminal α-linked mannose or biantennary N-glycans with multiple extensions, core 2 o-glycans, bisecting GlcNAc preferred with type 2 LacNAc, as well as α-linked fucose in specific linkages (Figure 1, Table 1 and Table S1). The observed lectin binding (e.g., by RCA I, ConA, ABL, PHA-E, and AAL; see Table 1) suggests the presence or accessibility of corresponding glycan motifs. However, due to the limited specificity of plant lectins and the influence of glycan context and conformation, these findings reflect the probable occurrence of terminal glycan features rather than complete structural elucidation. All other glycan epitopes showed intermediate signal intensities, further emphasizing the complexity and heterogeneity of surface glycosylation in equine neutrophils.

**Figure 2:**
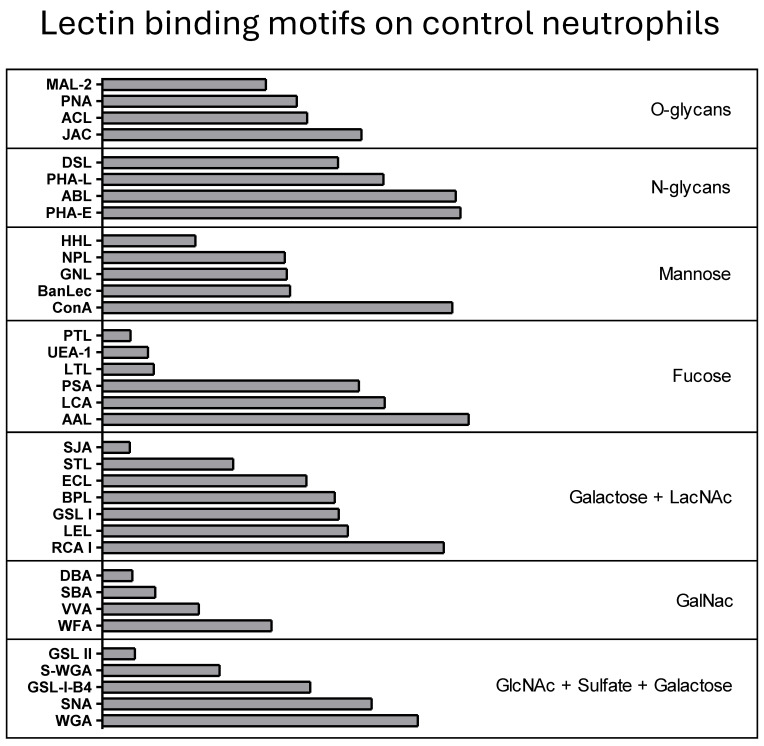
Lectin binding motifs on neutrophils from healthy control horses (*n* = 7; except AAL, ABL, ACL, GSL II, STL, WGA: *n* = 6 and DSL, JAC, LCA, LEL, PSA, RCA, SNA: *n* = 5; data was obtained in six independent experiments). Binding intensity of all 35 biotinylated plant lectins used in flow cytometry assays is shown with bar graphs analogous to Figure 1. Lectins are grouped according to their binding motif and arranged from low binding to high binding intensity within each group.

For a better overview of surface glycans, lectins were grouped according to their glycan binding motifs, resulting in seven distinct groups [32] (Figure 2): lectins with O-glycan binding, N-glycan binding, mannose binding, fucose binding, terminal galactose and LacNAc binding, GalNAc binding and GlcNAc, and sulfate and galactose binding motifs.

### 3.2. Differentially Expressed Glycans on ERU Neutrophils

**Figure 3:**
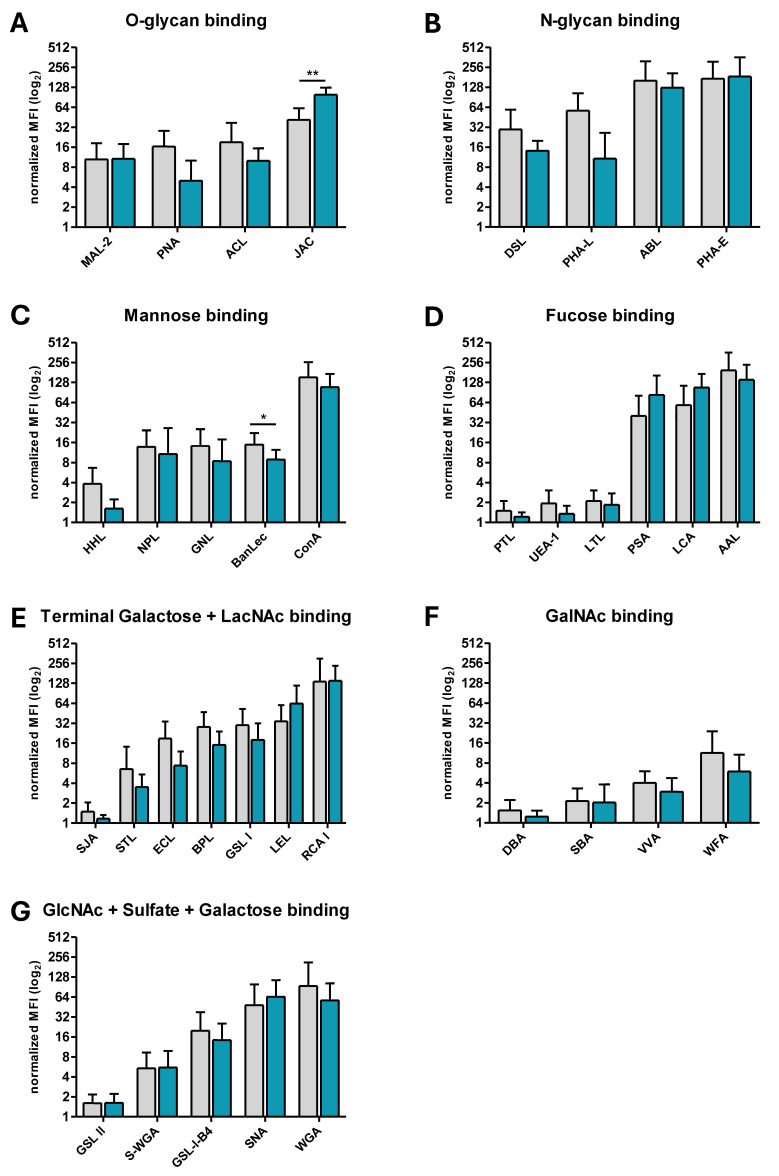
Surface glycosylation pattern of neutrophils from ERU cases (*n* = 11; except DBA, GSL II, HHL, LTL, MAL-2, NPL, PNA, UEA-1: *n* = 10 and ABL, DSL, JAC, LCA, LEL, PSA, PTL, RCA-I, SBA, SJA, SNA: *n* = 9) compared to controls (*n* = 7; except AAL, ABL, ACL, GSL II, STL, WGA: *n* = 6 and DSL, JAC, LCA, LEL, PSA, RCA, SNA: *n* = 5; data was obtained in six independent experiments). Binding intensities of the 35 biotinylated plant lectins used in flow cytometry assays are shown with bar graphs (gray: controls, blue: ERU) and are arranged according to the grouped binding motifs in Figure 2: (**A**) O-glycans with significant 2.4-fold increase in JAC reactive O-glycans on neutrophils from ERU cases (Student’s *t*-test with Welch’s correction, ** *p* = 0.0012), (**B**) N-glycans, (**C**) mannose with significant 1.7-fold decrease in BanLec reactive glycans on neutrophils from ERU cases (Mann–Whitney test, * *p* = 0.0163), (**D**) fucose, (**E**) galactose and LacNAc, (**F**) GalNac, and (**G**) GlcNAc and sulfate and galactose. Mean fluorescence intensity (MFI ± SD) of lectin binding was normalized to respective unstained cells per animal. Outliers were excluded via Grubb’s test. The *y*-axis is displayed on a log_2_ scale to account for differences in binding intensity across several orders of magnitude.

To investigate whether surface glycosylation of neutrophils is altered in equine recurrent uveitis (ERU), lectin binding profiles of neutrophils from ERU cases were compared to those from healthy controls. Thirty-three of the thirty-five lectins used for the identification of equine surface glycosylation showed no significantly changed binding (Figure 3B,D–G), whereas two lectins showed statistically significant differences in binding intensity between controls and ERU cases. These lectins belonged to the O-glycan-binding (Figure 3A) and mannose-binding groups (Figure 3C).

Five of the plant lectins used in the surface glycome screening were grouped as mannose binding: BanLec, ConA, GNL, HHL, and NPL (Figure 2 and Figure 3C). The lectin BanLec has binding sites for internal (α-1,3) glucosyl and mannosyl residues as well as reducing terminal (α 1,3) and α (1,6) glucosyl residues. HHL primarily detects terminally bound α-mannoses, which are also bound by ConA. However, ConA additionally detects biantennary N-glycans with multiple extensions. The plant lectins GNL and NPL preferably bind to α1-6-linked mannose, but an affinity for α1-3-linked mannose and for N-glycans with terminated short LacNAc has also been reported. The terminally bound α-mannoses detected by HHL were only detectable to a very small extent on the neutrophils in both comparison groups (Figure 1). ConA-bound structures were highly abundant on equine neutrophils (Figure 1). The glycan structures identified by BanLec, GNL, and NPL showed moderate surface abundance (Figure 1). Among the five mannose-binding lectins in this group, only the glycan structures containing internal α-(1,3)-linked glucose and mannose residues and reducing terminal (α 1,3) and α (1,6) glucosyl residues detected by BanLec showed significantly lower abundance on the cell surface of ERU neutrophils (1.7-fold, * *p* = 0.0163) (Figure 3C, Table 1).

Four of the plant lectins used for surface glycan screening could be used to detect O-glycans, namely ACL, JAC, MAL-2, and PNA (Figure 2 and Figure 3A). ACL recognizes core 1 and core 2 O-glycans and poly Lewis structures. JAC, a lectin with a high affinity for mucin-type O-glycans, also binds to Galβ1-3GalNAc structures but prefers core 1 and core 3 glycans, and even tolerates mono- and disialylation. In contrast, PNA only detects terminally accessible core 1 and core 2 O-glycans. The lectin MAL-2 interacts primarily with α 2-3-sialylated O-glycans. The O-glycans bound by ACL, JAC, MAL-2, and PNA could be detected on the cell surface with moderate to high abundance (Figure 1) with no significant differences between controls and ERU (Figure 3A, Table 1). In contrast, JAC-bound O-glycans showed a significantly increased abundance on neutrophils from ERU cases (2.4-fold, ** *p* = 0.0012) (Figure 3A, Table 1).

While JAC is commonly used as a tool to detect O-glycoproteins due to its affinity for galactosyl (β-1,3) N-acetylgalactosamine, its specificity has limitations. JAC does not bind α-linked N-acetylgalactosamine if the sugar is modified at the C6 position or capped by sialic acid [33]. Thus, while the observed increase reflects altered O-glycosylation, it may underestimate total mucin-type glycan complexity on ERU neutrophils. To evaluate JAC specificity, we performed lectin inhibition via its monosaccharide agonist galactose as well as sucrose and mannose (Figure S2). Pre-incubation of JAC with galactose caused strongly decreased binding to equine neutrophils, which did not occur after JAC inhibition with sucrose and mannose (Figure S2).

**Table 1:** Alternations in surface glycosylation of equine neutrophils sorted by *p*-value. Normalized mean fluorescence intensities (MFI) show differences in lectin binding to specific glycan structures on neutrophils from controls (*n* = 5–7) and ERU cases (*n* = 9–11). (a) Lectin acronym, (b) binding specificity according to Bojar et al. [32], Lescar et al. [34], Singh et al. [35], and Monsigny et al. [36], (c) normalized mean fluorescence intensity (MFI) measured for binding of lectins on granulocytes from controls and ERU cases, with standard deviation (SD), (d) *p*-value of differential lectin binding intensity in controls compared to ERU cases.

Acronym ^a^	Specificity ^b^	Norm. MFI ± SD ^c^		*p*-Value Ctr vs. ERU ^d^
		Controls	ERU	
PHA-E	bisecting GlcNac preferred with type 2 LacNAc	172.6 ± 136.9	185.4 ± 176.2	1.000
RCA I	terminal ß-galactose residues, with a preference for type 2 LacNAc	135.6 ± 164.8	139.4 ± 95.0	0.9638
S-WGA	Oligosaccarides containing terminal GlcNAc	5.4 ± 3.9	5.5 ± 4.2	0.9396
AAL	α-linked fucose; preferring (α-1,2)-linked fucose terminated structures on type 2 LacNAc	135.6 ± 71.8	139.8 ± 95.2	0.8802
STL	internal type 2 LacNAc as linear glycans; type 2 LacdiNAc; Chitin oligomers	3.9 ± 3.1	3.5 ± 1.9	0.8802
ABL	biantennary N-glycans terminating with ß-GlcNAc or LacNAc; core 2 O-glycans	108.7 ± 73.0	126.0 ± 82.3	0.6764
MAL-2	(α-2,3) sialylated O-glycans	10.5 ± 7.9	8.7 ± 3.5	0.6100
GSL II	α- or β-linked GlcNAc, preferring GlcNac capped LacNAc in multiantennary N-glycans	1.6 ± 0.6	1.5 ± 0.3	0.5944
SNA	(α-2,6) sialylated LacNAc or LacdiNAc	48.0 ± 50.6	64.5 ± 49.9	0.5746
SBA	Oligosaccharide structures with terminal α- or β-linked GalNAc	2.1 ± 1.2	2.2 ± 1.9	0.5220
WGA	multiple terminal N-acetylstructures; oligosaccharides containing terminal α- or β-linked GlcNac	52.8 ± 54.1	57.1 ± 45.2	0.5136
LTL	(α-1,3) linked fucose containing oligosaccharides	2.1 ± 0.9	1.6 ± 0.4	0.4927
GSL-I-B4	terminal α-galactose residues	19.9 ± 17.8	14.3 ± 11.1	0.4777
PTL	Carbohydrate structures containing (α-1,3) linked galactose and (α-1,2) linked fucose	1.5 ± 0.6	1.2 ± 0.2	0.4482
DSL	(β-1,4) and (β-1,6) linked GlcNAc oligomers, preferring chitobiose or chitotriose; branching structures with several type 2 LacNac repeats	18.4 ± 10.2	14.1 ± 5.8	0.4271
ConA	terminal α-linked mannose, biantennary N-glycans with multiple extensions	153.1 ± 105.8	108.6 ± 64.1	0.3450
DBA	terminal GalNAc-α1,3-GalNAc	1.5 ± 0.7	1.2 ± 0.2	0.3166
SJA	terminal poly- or multiantennary type 2 LacNAc or LacdiNAc; bloodgroup B	1.5 ± 0.6	1.2 ± 0.2	0.2804
WFA	Carbohydrate structures terminating in α- or β-linked GalNAc, terminal LacNAc	11.4 ± 12.5	5.9 ± 4.7	0.2766
VVA	terminal α- or β-linked GalNac, terminal ß-LacdiNAc	4.0 ± 2.0	2.9 ± 1.8	0.2716
ACL	core 1 and core 2 O-glycans; poly Lewis structures	12.6 ± 7.2	10.0 ± 5.4	0.2272
PSA	core fucose structures	40.0 ± 40.5	82.7 ± 80.0	0.2123
LEL	type 2 polyLacNac; type 2 LacdiNAc; Chitin oligomers	34.1 ± 25.8	63.6 ± 54.5	0.1978
LCA	Core fucose; terminal (α-1,2) mannose	58.0 ± 55.8	106.9 ± 64.3	0.1714
HHL	terminal α-mannose structures	3.8 ± 2.9	2.0 ± 0.6	0.1711
GSL I	α-GalNac residues and α-galactose residues	29.9 ± 22.7	18.0 ± 14.0	0.1600
UEA-1	(α-1,2) linked fucose to (β-1,4) linked galactose	1.9 ± 1.1	1.3 ± 0.4	0.1521
BPL	oligosaccharides with terminal β-linked galactose or β-linked GalNac	28.2 ± 18.8	15.1 ± 8.9	0.1282
ECL	terminal type 2 LacNAc or LacdiNAc	18.8 ± 15.1	7.3 ± 4.6	0.0993
NPL	terminal (α-1,6) and (α-1,3) mannose residues; N-glycans with terminated short LacNAc	13.8 ± 10.5	6.3 ± 4.6	0.0878
PHA-L	(ß-1,6)-branched N-glycans	56.9 ± 47.0	27.2 ± 19.6	0.0853
GNL	terminal (α-1,6) and (α-1,3) mannose residues; N-glycans with terminated short LacNAc	14.2 ± 11.2	8.2 ± 8.9	0.0700
PNA	core 1 and core 2 O-glycans	15.4 ± 11.1	5.0 ± 5.0	0.0636
BanLec	Internal (α-1,3) glucosyl- and mannosyl residues; reducing terminal (α-1,3) and (α-1,6) glucosyl residues	21.1 ± 17.8	8.8 ± 3.6	0.0163
JAC	core 1 and core 3 O-glycans; 3-substituents α-GalNAc	41.5 ± 20.3	98.9 ± 27.2	0.0012

### 3.3. BanLec-Bound Glycans Show Significantly Decreased Abundance on the Surface of ERU Neutrophils, Whereas JAC-Bound Glycans Are Significanty More Abundant

**Figure 4:**
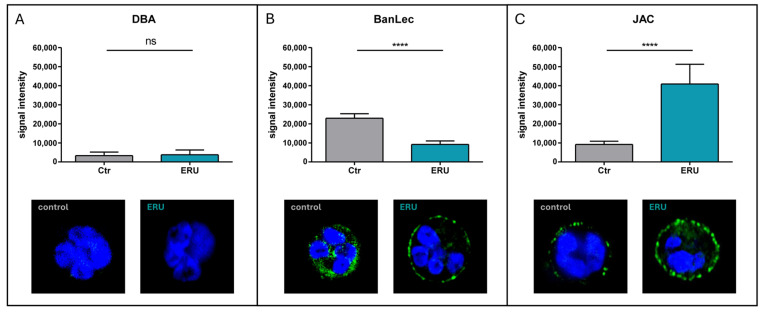
ERU-associated changes in neutrophil surface glycosylation (ERU: *n* = 3, controls: *n* = 3; data was obtained in five independent experiments). Immunocytochemistry analyses visualize binding intensity of each represented lectin to cell surface glycan structures (green). Cell nuclei were counterstained with DAPI (blue). Bar graphs display quantification of lectin signal intensity measured via immunocytochemisty (gray: controls, blue: ERU). (**A**) DBA-reactive glycans were not detected in either control or ERU cases since no lectin binding can be observed (no green visible) (Student’s *t*-test, ns). (**B**) BanLec-reactive glycans were significantly decreased on neutrophils from ERU cases reflected by decreased lectin binding (green) and corresponding quantification data shown in bar graphs (Student’s *t*-test, **** *p* < 0.0001). (**C**) JAC-reactive glycans were significantly increased on the surface of neutrophils from ERU cases as shown by increased lectin binding (green) and corresponding quantification data (Student’s *t*-test with Welch’s correction, **** *p* < 0.0001).

To explore these differences in more detail, we selected the significantly different surface glycans detected via Banana Lectin (BanLec; Mannose-binding) and Jacalin (JAC; O-glycan binding) for further validation and quantification with immunocytochemistry. Dolichos Biflorus Agglutinin (DBA) was used as a negative control since DBA-reactive glycans were absent in controls and ERU in the flow cytometry screening with all 35 plant lectins (Figure 1 and Figure 3). In the immunocytochemistry experiments, DBA-reactive glycans were also not detected, confirming the consistent lack of detectable terminal GalNAc-α1,3-GalNAc residues (Figure 4A).

BanLec-reactive structures, which are associated with internal α-1,3-glucosyl and mannosyl residues, visibly decreased in the immunocytochemistry experiments (Figure 4B). Quantification revealed a significant reduction in BanLec-reactive structures on the surface of ERU neutrophils compared to controls (**** *p* < 0.0001; Figure 4B). In contrast, JAC, which binds to galactosyl (β-1,3) N-acetylgalactosamine structures, showed significantly increased binding intensity to ERU neutrophils, which is clearly evident in the immunocytochemistry images (**** *p* < 0.0001; Figure 4C). These findings highlight surface glycan abundance differences on the cellular level, supporting the disease-associated redistribution of specific O-glycan and mannose motifs detected in our initial screening assays.

Together, the immunocytochemistry results confirm the selected changes in surface glycosylation identified by flow cytometry in the initial surface glycosylation screening assay, suggesting that ERU alters the surface glycan landscape of circulating neutrophils in a structure-specific manner.

### 3.4. JAC Lectin Pull-Down Reveals ERU-Associated O-Glycosylated Proteins

**Figure 5:**
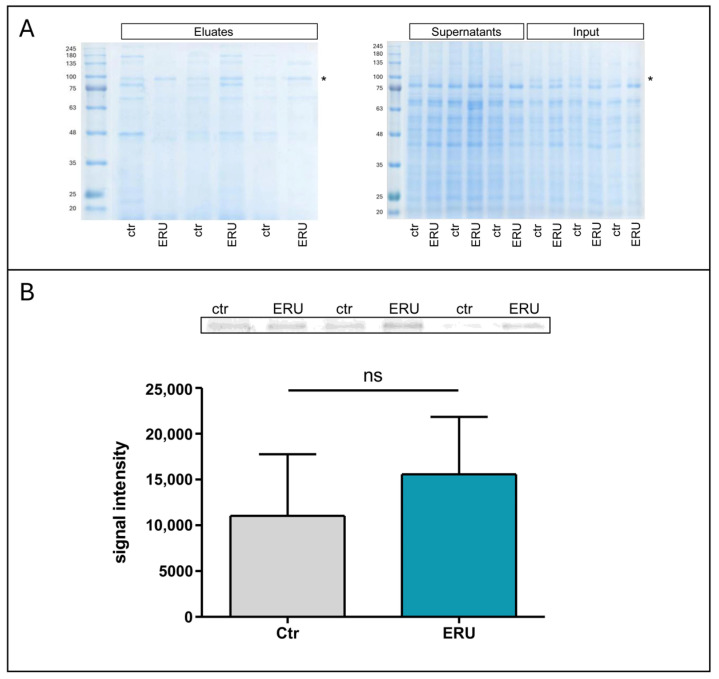
Proteins that were enriched via JAC lectin pull-down assay (controls: *n* = 3, ERU: *n* = 3; shown data were obtained in one experiment). (**A**) 1D-SDS-gels (10%) run with eluates from the JAC lectin pull-down assay showing bands at 94.5 kDa in all lanes (asterisk), indicating the molecular weight of O-glycosylated proteins bound by JAC in equine neutrophils (left), and 1D-SDS-gels (10%) run with supernatants from the JAC lectin pull-down assay and lysates that were used as input (right) show no or weak bands at 94.5 kDa (asterisk). (**B**) Statistical analysis of the 94.5 kDa band intensity in controls (gray) and ERU cases (blue). Data are represented as mean ± SD. Band intensities in ERU cases were 1.4-fold higher compared to controls (Mann–Whitney test, ns *p* > 0.05). The band of interest is shown more detailed in greyscale above the bar chart.

**Figure 6:**
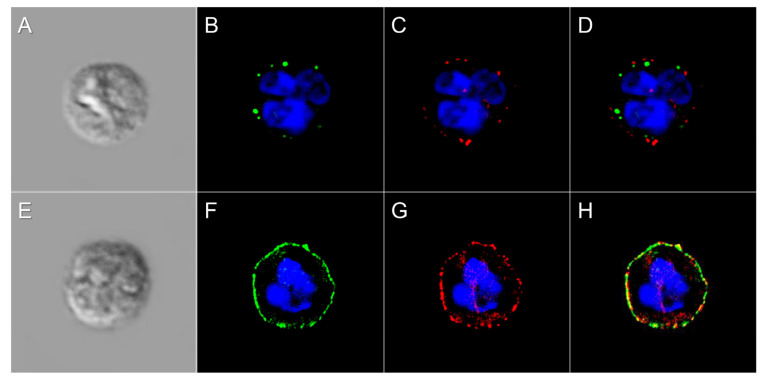
Representative images of JAC and anti-Integrin beta-2 binding on the surface of neutrophils from controls (**A**–**D**) and ERU horses (**E**–**H**) (controls: *n* = 1, ERU: *n* = 2; data were obtained in one experiment). (**A**,**E**) DIC image of equine neutrophils, (**B**,**F**) JAC staining on the cell surface indicating O-glycosylation (green), (**C**,**G**) anti-Integrin beta-2 staining indicating the presence of Integrin beta-2 (red), (**D**,**H**) overlay of JAC staining (green) and anti-Integrin beta-2 staining (red) shows colocalization of JAC-bound O-glycans with Integrin beta-2 (yellow). JAC and anti-Integrin beta-2 show increased binding to neutrophils from ERU cases (**D**–**F**) compared to controls (**A**–**C**). Cell nuclei were stained with DAPI (blue).

To further characterize the increased JAC-reactivity observed in ERU neutrophils, we performed a JAC lectin pull-down assay using agarose bound JAC to enrich for surface-accessible O-glycoproteins. SDS-PAGE analysis of lysates, supernatants, and eluates revealed multiple protein bands, among which a prominent band at 94.5 kDa was consistently observed in both ERU cases and controls (Figure 5A). This band appeared more intense in ERU, and densitometric analysis confirmed a 1.4-fold higher signal in ERU neutrophils (Figure 5B), although the difference did not reach statistical significance. To clarify whether this band might correspond to proteins with increased overall abundance in ERU neutrophils, we searched previously published proteome data for candidate proteins that (i) fall within the 90–100 kDa molecular weight range, (ii) are located on the cell surface, (iii) possess predicted O-glycosylation sites, and (iv) were detected in all six mass spectrometry samples. We then filtered for proteins with an abundance ratio ≥1.1 in ERU cases (Table 2). It should be noted that this approach specifically identifies proteins with increased abundance in ERU neutrophils but does not assess changes in glycosylation per protein molecule. Therefore, the identified candidates represent surface proteins that are not only potentially O-glycosylated but also more abundant in ERU and could thus account for the observed increase in JAC binding. Among the five proteins fulfilling all criteria, CUB domain-containing protein 1 and Integrin beta-2 were approximately 2-fold more abundant in ERU cases, as displayed in flow cytometry analysis, and fell within the relevant molecular weight range. These proteins are plausible contributors to the increased JAC-reactive band at 94.5 kDa. Subsequent immunocytochemistry analysis revealed a noticeable increase in Integrin beta-2 on ERU neutrophils compared to controls (Figure 6C,G), as well as an increased presence of JAC-bound O-glycans (Figure 6B,F). Further, we detected colocalization of JAC-bound surface glycosylation and Integrin beta-2 (Figure 6D,H), underlining Integrin beta-2 as a candidate O-glycosylated protein on neutrophils with increased abundance and/or O-glycosylation in ERU.

## 4. Discussion

Neutrophils are highly abundant cells of the innate immune system with a wide variety of effector functions and implications in diseases. Neutrophil activation status is mirrored in the cell surface protein landscape and can be presented through protein abundance changes as well as post-translational modifications (PTM) of surface proteins [2]. One of the crucial PTMs during cell activation is glycosylation, which can shape immune response through impact on cell–cell interactions such as antigen presentation and modification of signal transduction [37,38].

Surface glycosylation of neutrophils has previously been described for specific proteins, for single glycans or in the context of certain diseases. For example, it is well-described on human neutrophils that toll-like receptor 4, a member of one of the most important cell receptor families of the innate immune system, shows several N-glycosylation sites [39,40]. Further, N-glycome profiling of isolated neutrophil granules and vesicular populations has been described for human neutrophils [41]. Neutrophil granules and vesicles contain a large amount of highly glycosylated glycoproteins. Once neutrophils are activated, as is the case in equine recurrent uveitis (ERU) [22,42], they can express these intracellularly stored glycoproteins on the cell surface to modulate their immune response [43]. Of note, protein biosynthesis is low in neutrophils [44], meaning that the majority of neutrophil glycoproteins is already present within the cell and can be re-located to the cell surface in the course of activation. Knowledge of the neutrophil surface glycosylation landscape, independent of specific proteins, glycan moieties, or disease, is scarce to date, especially in large animal models. Therefore, we investigated the cell surface of equine neutrophils for the presence of a variety of different glycans and are the first to describe a comprehensive surface glycome for equine neutrophils. To achieve this, we used 35 different plant lectins, each with a specific glycan structure as binding target. The resulting lectin binding pattern underlines the heterogeneity of surface glycosylation on equine neutrophils. To date, the full spectrum of neutrophil surface glycosylation remains largely unknown. Given that mammals express an immense diversity of surface glycans, our study provides a valuable initial overview of the equine neutrophil surface glycome, based on the binding specificities of 35 plant lectins. These important aspects of the neutrophil surface glycan profile in its “healthy” state have not been described before and are useful as a basis for further investigations on the composition of surface-exposed glycan moieties on neutrophils. Since protein glycosylation impacts neutrophil effector functions [43], we were especially interested in possible changes in surface glyco-signatures of neutrophils from horses with ERU. ERU is a painful, sight-threatening disease in horses, with a remitting–relapsing character and increasingly severe inflammatory attacks over time [45]. While its exact etiology remains unclear, ongoing research continues to explore potential triggers, biomarkers for pathophysiology, and causative treatment options. Described as primarily driven by autoreactive CD4+ T cells, this cell type dominates among the immune cell populations found in ocular infiltrates in ERU [46,47]. However, pure neutrophil infiltrates can be observed in spontaneous ERU and interphotoreceptor retinoid-binding protein-induced cases, even in early stages of uveitis [21,48]. In addition, circulating neutrophils show an activated phenotype in ERU, even between uveitic attacks [22,49] and more readily release neutrophil extracellular traps, which have been shown to associate with disease severity [25,50], pointing to active involvement in ERU pathogenesis. Besides its importance for veterinary medicine, ERU is a promising model for autoimmune-mediated recurrent uveitis in humans, given notable parallels in clinical and pathophysiological characteristics as well as immune system composition and function [11,14,51,52]. The predominance of neutrophils among the blood-derived leukocyte population in both humans and horses makes the horse especially interesting for investigations on early disease-initiating events which may be neutrophil-mediated. This allows for better extrapolation of findings to human disease pathomechanisms as opposed to rodent models for autoimmune uveitis, which have a very different immune system organization [53]. Although autoimmune uveitis is described as T-cell driven, the active involvement neutrophils in disease pathogenesis is becoming increasingly evident as neutrophils were recently suggested to have a regulatory effect on T cells via the formation of neutrophil extracellular traps [54]. These observations in humans, rodent models, and horses suggest neutrophils to be active participants in shaping adaptive immune responses in AU and ERU, which emphasizes the role of the innate immune system in this sight-threatening disease.

A few of the plant lectins used in our study, including HHL, LTL, PSA, PTL, UEA-1, SJA, DBA, SBA, VVA, and GSL II, showed weak to no binding to equine neutrophil surface glycans (Figure 1, Table 1). This indicates that said surface glycans may not be part of the equine neutrophil surface glycome. To determine whether this originates in absence or in poor lectin-accessibility of glycans [55], further analyses can be performed under different experimental conditions that determine how the glycan-binding sites are presented [56]. Most of the glycan moieties that we detected on the surface of neutrophils from healthy horses were similarly abundant in neutrophils of ERU cases. For example, core fucosylation—a type of N-glycosylation, catalyzed by fucosyltransferase 8, where fucose is attached to the N-glycan through an α-1,6-linkage [57]—which we detected via the plant lectins AAL, LCA, and PSA, showed equally strong presence on the surface of neutrophils from controls and ERU cases (Figure 1 and Figure 3, Table 1). Interestingly, surface fucosylation seems to specifically change on different leukocyte subtypes in other autoimmune diseases. For instance, increased core fucosylation can be observed on the surface of activated T cells from patients with systemic lupus erythematosus [6], indicating that defined glycan alterations specifically depend on cell type and disease. In humans, core fucosylation can be found in every subtype of neutrophil granules, with a particularly high abundance in azurophil granules [58], but to date there is no implication of core fucosylation on neutrophils in autoimmune disease pathogenesis.

We previously described an altered glycosylation profile on other cells in the context of ERU: In retinal Müller glia cells, the main macroglia cells of the retina with a pivotal role in maintaining retinal function and integrity, we could demonstrate a loss of α2-3- and α2-6-linked terminal sialic acids in the uveitic state via lectin-binding (MAL-2, SNA, WGA) in immunohistochemistry analyses of equine retinae [59]. These terminal sialic acids were also identified on neutrophils in the present study, but with equal abundance in health and disease (Figure 1 and Figure 3, Table 1). This suggests that terminal sialic acids may belong to the basic glycan signature on neutrophils, which remains unaltered on activated neutrophils in ERU. Interestingly, we identified two other glycan structures that were significantly altered on the neutrophil surface in ERU: glycans that are bound by the plant lectin BanLec and glycans that are bound by JAC (Figure 3, Table 1). Since neutrophils continue to emerge as a heterogeneous leukocyte population with a variety of finely tuned effector functions [60,61,62], which may also be influenced by the nature of their surface glycosylation, particularly in different inflammatory environments and with regard to their activation status, these significant changes in the surface glycome signature are especially interesting in the context or ERU.

Notably, we detected a significant decrease in mannose or glucose linked N-glycans through binding to the plant lectin Banana Lectin (BanLec) on the surface of neutrophils from ERU cases in flow cytometry and immunochemistry (Table 1, Figure 3C and Figure 4B), which is a new finding on equine neutrophils. Interestingly, we detected that binding to ConA, GNL, und NPL was also decreased in ERU cases, but not significantly. This may be explained by their similar binding specificity, since these lectins can be grouped as mannose-binding, whereas BanLec additionally binds internal (α-1,3) glucosyl residues and reducing terminal (α 1,3) and α (1,6) glucosyl residues [32,35]. Mannose linked N-glycans are described in healthy human neutrophil granules [58] but changed surface abundance has not been described for neutrophils in autoimmune diseases so far. In contrast, the plant lectin JAC was the only lectin with significantly elevated binding to neutrophils from ERU cases (Table 1, Figure 3A and Figure 4C). JAC has a highly specific binding affinity for core 1 and core 3 O-glycans, and it is therefore widely used as a reliable marker for O-glycosylation [32,33,63,64]. Interestingly, the observed binding alteration in ERU could not be detected for the other lectins with O-glycan binding specificity, such as PNA, ACL, and MAL-2. JAC predominantly binds to core 1 and core 3 O-glycans, whereas ACL and PNA are also able to bind core 1 and core 2 O-glycans, but not to the core 3 O-glycans, and MAL-2 only recognizes α2,3-sialylated O-glycans [32]. This emphasizes the unique selectivity of JAC to bind certain types of O-glycans on neutrophils of ERU cases and makes it a promising biomarker candidate. Although our study is an important first step towards elucidating disease-associated changes in neutrophil surface O-glycosylation, further experimental approaches—such as enzymatic trimming of O-glycans, glycomic mass spectrometry, or site-specific analysis of glycoproteins—are needed to provide more detailed structural insight. Divergent O-glycosylation on neutrophils in autoimmune uveitis is a novel discovery that has not been investigated so far. The increased presence of O-glycans may reflect the presence of a generally increased abundance of JAC-specific O-glycosylation on the neutrophil membrane or may occurdue to increased abundance of specific O-glycosylated proteins. Distinguishing between these two possibilities is critical, as they imply fundamentally different modes of regulation—one at the level of glycan processing and the other at the level of protein expression and turnover—that may influence neutrophil activation states and effector functions in different ways. Regardless of the origin, the functional meaning of the increased O-glycosylation on neutrophils in ERU remains unclear and needs further in-depth investigations.

To further characterize the specific O-glycosylation which we identified via Jacalin (JAC) binding, we aimed at finding potentially O-glycosylated proteins targeted by JAC. To achieve this, we enriched O-glycoproteins via JAC pull-down and detected several different protein bands. One particular band was present in all JAC pull-down eluates, at a molecular weight of 95.4 kDa. This specific protein band was more intense in ERU (Figure 5). After reinvestigating neutrophil proteomics data, filtering for the defined criteria molecular weight 90–100 kDa, cell surface proteins, possibility of O-glycosylation, identification in all measured samples, as well as higher abundance in ERU, we found two possible membrane protein candidates as anchors for O-glycosylation: CUB domain-containing protein 1 and Integrin beta-2 (Table 2). Although these two candidate proteins may be a promising starting point for glycoprotein biomarker discovery, we need to keep in mind that the identification of these proteins is based on an indirect approach, conducted by molecular weight comparison of pull-down data and proteomics analyses. Moreover, other O-glycosylated proteins may also be present, even if not (yet) identified, and more in-depth investigations are needed to determine a more comprehensive O-glycan surface landscape on equine neutrophils with potential alterations in disease.

CUB domain-containing protein 1 is an O-glycosylated protein with a molecular weight of 93 kDa. It is located at the cell membrane, characterized as a single-pass membrane protein, and is 2.2-fold more abundant in proteomics data from ERU neutrophils. CUB domain-containing protein 1 is a ligand for CD6, which is a type I transmembrane glycoprotein expressed by T-lymphocytes and has been described to be involved in the pathophysiology of autoimmune-mediated diseases as multiple sclerosis and rheumatoid arthritis [65]. Interestingly, CUB domain-containing protein 1is highly expressed by retinal pigment epithelial cells (RPE), and CUB domain-containing protein 1-CD6 interaction has been shown to facilitate T cell transmigration through the RPE monolayer in mice, resulting in uveitis [66]. The role of CUB domain-containing protein 1 in neutrophils has not previously been investigated. However, regarding its implication in induced uveitis in the mouse via the CUB domain-containing protein 1-CD6 axis, the presence and increased abundance of CUB domain-containing protein 1 in neutrophils is in alignment with the generally more activated equine neutrophils in ERU [22].

Integrin beta-2is a protein with predicted O-gylcosylation, which was calculated via NetOGlyc 4.0 [31]. It has a molecular weight of 94.8 kDa, which is a good fit to the protein bald precipitated with JAC at 95.4 kDa; it is located at the cell membrane and was detected in the neutrophil proteome with 1.9-fold higher abundance in ERU. Integrin beta-2 is a member of the transmembrane receptor family composed of non-covalently associated heterodimers formed by one α and one β subunit. In neutrophils, integrin beta-2 mediates firm adhesion to the vascular endothelium, a prerequisite for transendothelial migration into inflamed tissues. Additionally, it facilitates key effector functions including chemotaxis, phagocytosis, and the formation of immunological synapses, thereby playing a central role in innate immune responses [67]. Due to the strong binding of the plant lectin JAC to neutrophils of ERU horses, we showed increased O-glycosylation in the diseased state, which might be associated with increased Integrin beta-2 abundance. Although we could show a higher abundance of Integrin beta-2 on neutrophils in ERU cases and colocalization of Integrin beta-2 and JAC binding on equine neutrophils via immunocytochemistry, more investigations are needed to confirm O-glycosylation of this candidate protein. Deviant glycan moieties on the Integrin beta-2 receptor molecule, such as terminal fucose and GlcNAc glycans as well as sialylated glycans, play a role in regulating neutrophil function such as transepithelial migration, degranulation, apoptosis, and superoxide generation in inflammatory mucosal diseases [68,69,70]. This implies that deviant O-glycosylation on the Integrin beta-2 receptor of neutrophils can have an impact in the regulation of inflammation. Since Integrin beta-2 bound glycans have been described as potential targets for selective manipulation of neutrophil function [69], the possibility of O-glycosylated Integrin beta-2 to serve as a biomarker for ERU merits further assessment.

## 5. Conclusions

In this study, we provide the first characterization of the equine neutrophil surface glycome, revealing a largely conserved glycosylation profile across healthy horses and horses suffering from equine recurrent uveitis (ERU), a spontaneous equine model for autoimmune uveitis. Despite these similarities, we detected aberrant glyco-signatures in ERU, especially through increased binding of the plant lectin Jacalin, which points to increased O-glycosylation in the diseased state. Alignment with previously published neutrophil proteomic data suggests that the surface proteins Integrin beta-2 and CUB domain-containing protein 1 may serve as possible O-glycosylated protein biomarker candidates. While additional studies are required to fully disentangle the relative contributions of glycan modifications or glycoprotein expression, our data demonstrate disease-associated alterations in the neutrophil surface glycome in equine autoimmune uveitis.

## Figures and Tables

**Table 2:** Proteins of control and ERU neutrophils identified in all samples (6 of 6) of previously published proteome data, filtered after for molecular weight between 90 and 100 kDa, predicted O-glycosylation sites, and higher abundance (≥1.1) in ERU cases compared to controls. All listed proteins are located on the cell surface. Potential O-glycosylated JAC targets are highlighted in bold. (a) Protein name. (b) Name of the corresponding gene. (c) Protein accession number according to the Ensembl horse database (Version 75, www.ensembl.org (accessed on 2 July 2025)). (d) Ratio of protein abundance differences in ERU cases compared to controls. (e) Molecular weight of listed proteins according to uniprot database (https://www.uniprot.org (accessed on 2 July 2025)).

Protein ^a^	Gene Name ^b^	Accession Number ^c^	Ratio ERU/Controls ^d^	Molecular Weight ^e^
CUB Domain Containing Protein 1	* CDCP1 *	ENSECAP00000022716	2.2	93.0
Integrin, Beta 2	* ITGB2 *	ENSECAP00000021220	1.9	94.8
Adhesion G Protein-Coupled Receptor E5	*ADRE5*	ENSECAP00000008739	1.4	90.7
Oxysterol Binding Protein-Like 8	*OSBPL8*	ENSECAP00000013659	1.1	95.6
Glucosidase, alpha	*GANAB*	ENSECAP00000012762	1.1	96.3

## Data Availability

The raw data supporting the conclusions of this article will be made available by the authors on request. The raw proteome data have been deposited to the ProteomeXchange Consortium via the PRIDE repository with the dataset identifier: PXD013648 [30].

## Supplementary Materials

The following supporting information can be downloaded at: https://www.mdpi.com/article/10.3390/biom15101444/s1, Table S1: lectin binding intensity raw data from flow cytometry-based screening analyses with 35 plant lectins; Figure S1: Graph for molecular weight calculation of precipitated proteins; Figure S2: JAC inhibition via monosaccharides. Original images: RawData ICC and RawData WB.

## Abbreviations

The following abbreviations are used in this manuscript:

PTM	Post-translational Modifications
AU	Autoimmune Uveitis
ERU	Equine Recurrent Uveitis
RT	Room Temperature
PBS	Phosphate-buffered Saline
NaCl	Sodium Chloride
AAL	Aleuria Aurantia Lectin
ABL	Agaricus Bisporus Lectin
ACL	Amaranthus Caudatus Lectin
BanLec	Banana Lectin
BPL	Bauhinia Purpurea Lectin
ConA	Concanavalin A
DBA	Dolichos Biflorus Agglutinin
DSL	Datura Stramonium Lectin
ECL	Erythrina Cristagalli Lectin
GNL	Galanthus Nivalis Lectin
GSL I	Griffonia Simplicifolia Lectin I
GSL-I-B4	Griffonia Simplicifolia Lectin I Isolectin B4
GSL II	Griffonia Simplicifolia Lectin II
HHL	Hippeastrum Hybrid Lectin
JAC	Jacalin
LCA	Lens Culinaris Agglutinin
LEL	Lycopersicon Esculentum Lectin
LTL	Lotus Tetragonolobus Lectin
MAL-2	Maackia Amurensis Lectin II
NPL	Narcissus Pseudonarcissus Lectin
PHA-E	Phaseolus Vulgaris Erythroagglutin
PHA-L	Phaseolus Vulgaris Leucoagglutinin
PNA	Peanut Agglutinin
PSA	Pisum Sativum Agglutinin
PTL	Psophocarpus Tetragonolobus Lectin
RCA I	Ricinus Communis Agglutitin I
SBA	Soybean Agglutinin
SJA	Sophora Japonica Agglutinin
SNA	Sambucus Nigra Lectin
STL	Solanum Tuberosum Lectin
S-WGA	Succinylated Wheat Germ Agglutinin
UEA-1	Ulex Europaeus Agglutinin I
VVA	Vicia Villosa Lectin
WFA	Wisteria Floribunda Lectin
WGA	Wheat Germ Agglutinin
PFA	Paraform aldehyde
MFI	Mean Fluorescence Intensity
DAPI	4′, 6-Diamidin-2-phenylindol
KS	Kolmogorov–Smirnov Test
*CDCP1*	CUB Domain-containing Protein 1
*ITGB2*	Integrin β2
RA	Rheumatoid Arthritis
RPE	Retinal Pigment Epithelial Cells

## References

[1] de Haan N., Pucic-Bakovic M., Novokmet M., Falck D., Lageveen-Kammeijer G., Razdorov G., Vuckovic F., Trbojevic-Akmacic I., Gornik O., Hanic M. (2022). Developments and perspectives in high-throughput protein glycomics: Enabling the analysis of thousands of samples. Glycobiology.

[2] He M., Zhou X., Wang X. (2024). Glycosylation: Mechanisms, biological functions and clinical implications. Signal Transduct. Target. Ther..

[3] Marrero Roche D.E., Chandler K.B. (2024). Clinical glycoprotein mass spectrometry: The future of disease detection and monitoring. J. Mass Spectrom..

[4] Schjoldager K.T., Narimatsu Y., Joshi H.J., Clausen H. (2020). Global view of human protein glycosylation pathways and functions. Nat. Rev. Mol. Cell Biol..

[5] Kissel T., Toes R.E.M., Huizinga T.W.J., Wuhrer M. (2023). Glycobiology of rheumatic diseases. Nat. Rev. Rheumatol..

[6] Szabo E., Farago A., Bodor G., Gemes N., Puskas L.G., Kovacs L., Szebeni G.J. (2024). Identification of immune subsets with distinct lectin binding signatures using multi-parameter flow cytometry: Correlations with disease activity in systemic lupus erythematosus. Front. Immunol..

[7] Sahin F., Kaya Z.Z., Serteser M., Ozturk H.U., Baykal A.T. (2025). Glycan profiling of multiple sclerosis oligoclonal bands with MALDI-TOF. Anal. Methods.

[8] Huang J., Wang X., Li N., Fan W., Li X., Zhou Q., Liu J., Li W., Zhang Z., Liu X. (2024). YY1 Lactylation Aggravates Autoimmune Uveitis by Enhancing Microglial Functions via Inflammatory Genes. Adv. Sci..

[9] Wiedemann C., Amann B., Degroote R.L., Witte T., Deeg C.A. (2020). Aberrant Migratory Behavior of Immune Cells in Recurrent Autoimmune Uveitis in Horses. Front. Cell Dev. Biol..

[10] Peng X., Li H., Zhu L., Zhao S., Li Z., Li S., Wu D., Chen J., Zheng S., Su W. (2024). Single-cell sequencing of the retina shows that LDHA regulates pathogenesis of autoimmune uveitis. J. Autoimmun..

[11] Deeg C.A., Hauck S.M., Amann B., Pompetzki D., Altmann F., Raith A., Schmalzl T., Stangassinger M., Ueffing M. (2008). Equine recurrent uveitis--a spontaneous horse model of uveitis. Ophthalmic Res..

[12] Gilger B.C. (2018). Immune Relevant Models for Ocular Inflammatory Diseases. ILAR J..

[13] Soth R., Hoffmann A.L.C., Deeg C.A. (2024). Enhanced ROS Production and Mitochondrial Metabolic Shifts in CD4(+) T Cells of an Autoimmune Uveitis Model. Int. J. Mol. Sci..

[14] Malalana F., Stylianides A., McGowan C. (2015). Equine recurrent uveitis: Human and equine perspectives. Vet. J..

[15] Gerding J.C., Gilger B.C. (2016). Prognosis and impact of equine recurrent uveitis. Equine Vet. J..

[16] Li Z., Liu X., Li Z., Xiao Z., Chen G., Li Y., Huang J., Hu Y., Huang H., Zhu W. (2025). *STING* Deficiency Promotes Th17-Like Tfh to Aggravate the Experimental Autoimmune Uveitis. Investig. Ophthalmol. Vis. Sci..

[17] Barfusser C., Wiedemann C., Hoffmann A.L.C., Hirmer S., Deeg C.A. (2021). Altered Metabolic Phenotype of Immune Cells in a Spontaneous Autoimmune Uveitis Model. Front. Immunol..

[18] Caspi R.R., Chan C.C., Fujino Y., Najafian F., Grover S., Hansen C.T., Wilder R.L. (1993). Recruitment of antigen-nonspecific cells plays a pivotal role in the pathogenesis of a T cell-mediated organ-specific autoimmune disease, experimental autoimmune uveoretinitis. J. Neuroimmunol..

[19] Kerr E.C., Copland D.A., Dick A.D., Nicholson L.B. (2008). The dynamics of leukocyte infiltration in experimental autoimmune uveoretinitis. Prog. Retin. Eye Res..

[20] Kerr E.C., Raveney B.J., Copland D.A., Dick A.D., Nicholson L.B. (2008). Analysis of retinal cellular infiltrate in experimental autoimmune uveoretinitis reveals multiple regulatory cell populations. J. Autoimmun..

[21] Deeg C.A., Kaspers B., Gerhards H., Thurau S.R., Wollanke B., Wildner G. (2001). Immune responses to retinal autoantigens and peptides in equine recurrent uveitis. Investig. Ophthalmol. Vis. Sci..

[22] Weigand M., Hauck S.M., Deeg C.A., Degroote R.L. (2021). Deviant proteome profile of equine granulocytes associates to latent activation status in organ specific autoimmune disease. J. Proteom..

[23] Reno F., Pagano C.A., Bignotto M., Sabbatini M. (2025). Neutrophil Heterogeneity in Wound Healing. Biomedicines.

[24] Sheahan B.J., Schubert A.G., Schubert W., Sheats M.K., Schnabel L.V., Gilbertie J.M. (2025). Equine neutrophils selectively release neutrophil extracellular traps in response to chemical and bacterial agonists. Front. Vet. Sci..

[25] Fingerhut L., Ohnesorge B., von Borstel M., Schumski A., Strutzberg-Minder K., Morgelin M., Deeg C.A., Haagsman H.P., Beineke A., von Kockritz-Blickwede M. (2019). Neutrophil Extracellular Traps in the Pathogenesis of Equine Recurrent Uveitis (ERU). Cells.

[26] Maier-Begandt D., Alonso-Gonzalez N., Klotz L., Erpenbeck L., Jablonska J., Immler R., Hasenberg A., Mueller T.T., Herrero-Cervera A., Aranda-Pardos I. (2024). Neutrophils-biology and diversity. Nephrol. Dial. Transplant..

[27] Gilger B.C., Michau T.M. (2004). Equine recurrent uveitis: New methods of management. Vet. Clin. N. Am. Equine Pract..

[28] Schneider C.A., Rasband W.S., Eliceiri K.W. (2012). NIH Image to ImageJ: 25 years of image analysis. Nat. Methods.

[29] Kang D.H., Gho Y.S., Suh M.K., Kang C.H. (2002). Highly sensitive and fast protein detection with coomassie brilliant blue in sodium dodecyl sulfate-polyacrylamide gel electrophoresis. B Korean Chem. Soc..

[30] Perez-Riverol Y., Csordas A., Bai J., Bernal-Llinares M., Hewapathirana S., Kundu D.J., Inuganti A., Griss J., Mayer G., Eisenacher M. (2019). The PRIDE database and related tools and resources in 2019: Improving support for quantification data. Nucleic Acids Res..

[31] Steentoft C., Vakhrushev S.Y., Joshi H.J., Kong Y., Vester-Christensen M.B., Schjoldager K.T., Lavrsen K., Dabelsteen S., Pedersen N.B., Marcos-Silva L. (2013). Precision mapping of the human O-GalNAc glycoproteome through SimpleCell technology. EMBO J..

[32] Bojar D., Meche L., Meng G., Eng W., Smith D.F., Cummings R.D., Mahal L.K. (2022). A Useful Guide to Lectin Binding: Machine-Learning Directed Annotation of 57 Unique Lectin Specificities. ACS Chem. Biol..

[33] Tachibana K., Nakamura S., Wang H., Iwasaki H., Tachibana K., Maebara K., Cheng L., Hirabayashi J., Narimatsu H. (2006). Elucidation of binding specificity of Jacalin toward O-glycosylated peptides: Quantitative analysis by frontal affinity chromatography. Glycobiology.

[34] Lescar J., Loris R., Mitchell E., Gautier C., Chazalet V., Cox V., Wyns L., Pérez S., Breton C., Imberty A. (2002). Isolectins I-A and I-B of *Griffonia*(*Bandeiraea*) simplicifolia: Crystal structure of metal-free GS I-B_4_ and molecular basis for metal binding and monosaccharide specificity. J. Biol. Chem..

[35] Singh S.S., Devi S.K., Ng T.B. (2014). Banana lectin: A brief review. Molecules.

[36] Monsigny M., Sene C., Obrenovitch A., Roche A.C., Delmotte F., Boschetti E. (1979). Properties of succinylated wheat-germ agglutinin. Eur. J. Biochem..

[37] van Kooyk Y., Rabinovich G.A. (2008). Protein-glycan interactions in the control of innate and adaptive immune responses. Nat. Immunol..

[38] Wolfert M.A., Boons G.J. (2013). Adaptive immune activation: Glycosylation does matter. Nat. Chem. Biol..

[39] Ohnishi T., Muroi M., Tanamoto K. (2003). MD-2 is necessary for the toll-like receptor 4 protein to undergo glycosylation essential for its translocation to the cell surface. Clin. Diagn. Lab. Immunol..

[40] da Silva Correia J., Ulevitch R.J. (2002). MD-2 and TLR4 N-linked glycosylations are important for a functional lipopolysaccharide receptor. J. Biol. Chem..

[41] Kawahara R., Ugonotti J., Chatterjee S., Tjondro H.C., Loke I., Parker B.L., Venkatakrishnan V., Dieckmann R., Sumer-Bayraktar Z., Karlsson-Bengtsson A. (2023). Glycoproteome remodeling and organelle-specific N-glycosylation accompany neutrophil granulopoiesis. Proc. Natl. Acad. Sci. USA.

[42] Hoffmann A.L.C., Hauck S.M., Deeg C.A., Degroote R.L. (2022). Pre-Activated Granulocytes from an Autoimmune Uveitis Model Show Divergent Pathway Activation Profiles upon IL8 Stimulation In Vitro. Int. J. Mol. Sci..

[43] Ugonotti J., Chatterjee S., Thaysen-Andersen M. (2021). Structural and functional diversity of neutrophil glycosylation in innate immunity and related disorders. Mol. Asp. Med..

[44] Hughes V., Humphreys J.M., Edwards S.W. (1987). Protein synthesis is activated in primed neutrophils: A possible role in inflammation. Biosci. Rep..

[45] McMullen R.J., Fischer B.M. (2017). Medical and Surgical Management of Equine Recurrent Uveitis. Vet. Clin. N. Am. Equine Pract..

[46] Gilger B.C., Malok E., Cutter K.V., Stewart T., Horohov D.W., Allen J.B. (1999). Characterization of T-lymphocytes in the anterior uvea of eyes with chronic equine recurrent uveitis. Vet. Immunol. Immunopathol..

[47] Deeg C.A., Ehrenhofer M., Thurau S.R., Reese S., Wildner G., Kaspers B. (2002). Immunopathology of recurrent uveitis in spontaneously diseased horses. Exp. Eye Res..

[48] Deeg C.A., Thurau S.R., Gerhards H., Ehrenhofer M., Wildner G., Kaspers B. (2002). Uveitis in horses induced by interphotoreceptor retinoid-binding protein is similar to the spontaneous disease. Eur. J. Immunol..

[49] Degroote R.L., Schmalen A., Hauck S.M., Deeg C.A. (2023). Unveiling Differential Responses of Granulocytes to Distinct Immunostimulants with Implications in Autoimmune Uveitis. Biomedicines.

[50] Fingerhut L., Yucel L., Strutzberg-Minder K., von Kockritz-Blickwede M., Ohnesorge B., de Buhr N. (2022). Ex Vivo and In Vitro Analysis Identify a Detrimental Impact of Neutrophil Extracellular Traps on Eye Structures in Equine Recurrent Uveitis. Front. Immunol..

[51] Horohov D.W. (2015). The equine immune responses to infectious and allergic disease: A model for humans?. Mol. Immunol..

[52] Deeg C.A., Raith A.J., Amann B., Crabb J.W., Thurau S.R., Hauck S.M., Ueffing M., Wildner G., Stangassinger M. (2007). *CRALBP* is a highly prevalent autoantigen for human autoimmune uveitis. Clin. Dev. Immunol..

[53] Zschaler J., Schlorke D., Arnhold J. (2014). Differences in innate immune response between man and mouse. Crit. Rev. Immunol..

[54] Li Z., Li Z., Hu Y., Xie Y., Shi Y., Chen G., Huang J., Xiao Z., Zhu W., Huang H. (2025). Neutrophil extracellular traps potentiate effector T cells via endothelial senescence in uveitis. JCI Insight.

[55] Varki A. (2017). Biological roles of glycans. Glycobiology.

[56] Andre S., Kaltner H., Kayser K., Murphy P.V., Gabius H.J. (2016). Merging carbohydrate chemistry with lectin histochemistry to study inhibition of lectin binding by glycoclusters in the natural tissue context. Histochem. Cell Biol..

[57] Sun Y., Li X., Wang T., Li W. (2022). Core Fucosylation Regulates the Function of Pre-BCR, BCR and IgG in Humoral Immunity. Front. Immunol..

[58] Venkatakrishnan V., Dieckmann R., Loke I., Tjondro H.C., Chatterjee S., Bylund J., Thaysen-Andersen M., Karlsson N.G., Karlsson-Bengtsson A. (2020). Glycan analysis of human neutrophil granules implicates a maturation-dependent glycosylation machinery. J. Biol. Chem..

[59] Lorenz L., Amann B., Hirmer S., Degroote R.L., Hauck S.M., Deeg C.A. (2021). NEU1 is more abundant in uveitic retina with concomitant desialylation of retinal cells. Glycobiology.

[60] Silvestre-Roig C., Fridlender Z.G., Glogauer M., Scapini P. (2019). Neutrophil Diversity in Health and Disease. Trends Immunol..

[61] Ng L.G., Ostuni R., Hidalgo A. (2019). Heterogeneity of neutrophils. Nat. Rev. Immunol..

[62] Wigerblad G., Kaplan M.J. (2023). Neutrophil extracellular traps in systemic autoimmune and autoinflammatory diseases. Nat. Rev. Immunol..

[63] Jeyaprakash A.A., Katiyar S., Swaminathan C.P., Sekar K., Surolia A., Vijayan M. (2003). Structural basis of the carbohydrate specificities of jacalin: An X-ray and modeling study. J. Mol. Biol..

[64] Rubin J.R., Taylor S.K., Rudchenko S., Stojanovic M.N., Hess H. (2025). Single Molecule Kinetic Fingerprinting of Glycans on IgA1 Antibodies. Anal. Chem..

[65] Gurrea-Rubio M., Fox D.A., Castresana J.S. (2025). CD6 in Human Disease. Cells.

[66] Zhang L., Borjini N., Lun Y., Parab S., Asonye G., Singh R., Bell B.A., Bonilha V.L., Ivanov A., Fox D.A. (2022). *CDCP1* regulates retinal pigmented epithelial barrier integrity for the development of experimental autoimmune uveitis. JCI Insight.

[67] Wen L., Moser M., Ley K. (2022). Molecular mechanisms of leukocyte β2 integrin activation. Blood.

[68] Brazil J.C., Sumagin R., Cummings R.D., Louis N.A., Parkos C.A. (2016). Targeting of Neutrophil Lewis X Blocks Transepithelial Migration and Increases Phagocytosis and Degranulation. Am. J. Pathol..

[69] Brazil J.C., Kelm M., Lehoux S., Azcutia V., Cummings R.D., Nusrat A., Parkos C.A. (2020). Regulation of Neutrophil Function by Selective Targeting of Glycan Epitopes Expressed on the Integrin CD11b/CD18. FASEB J..

[70] Azcutia V., Kelm M., Fink D., Cummings R.D., Nusrat A., Parkos C.A., Brazil J.C. (2023). Sialylation regulates neutrophil transepithelial migration, CD11b/CD18 activation, and intestinal mucosal inflammatory function. JCI Insight.

