# Supplementary material for: Glycan Signatures on Neutrophils in an Equine Model for Autoimmune Uveitis
Source: Biomolecules. 2025 Oct 12;15(10):1444. doi: 10.3390/biom15101444 (PMC12562876; doi:10.3390/biom15101444)

# Figure 4

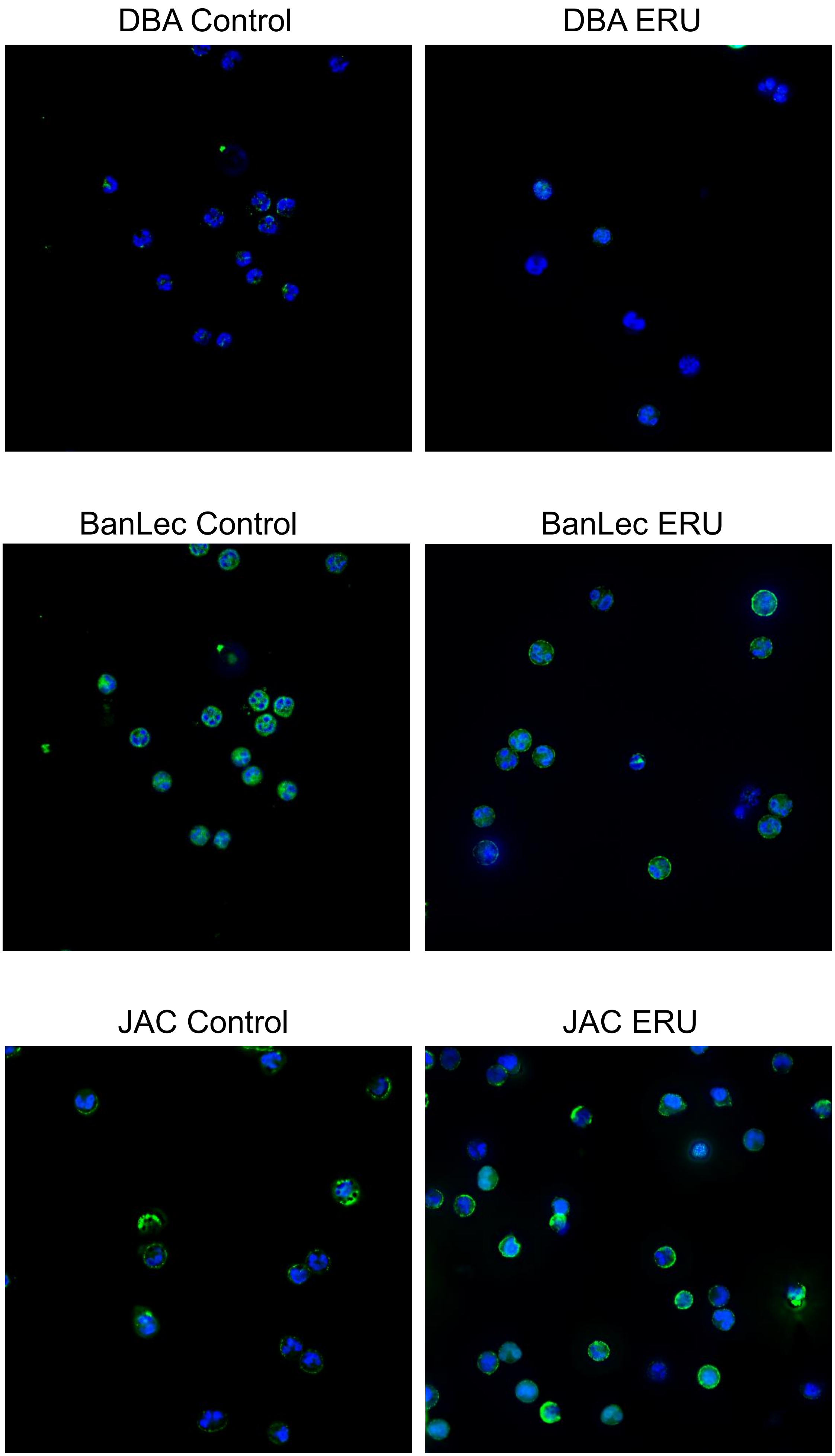
DBA Control
DBA ERU
BanLec Control
BanLec ERU
JAC Control
JAC ERU

# Figure 6

JAC + CD18 (ITGB2)
Control

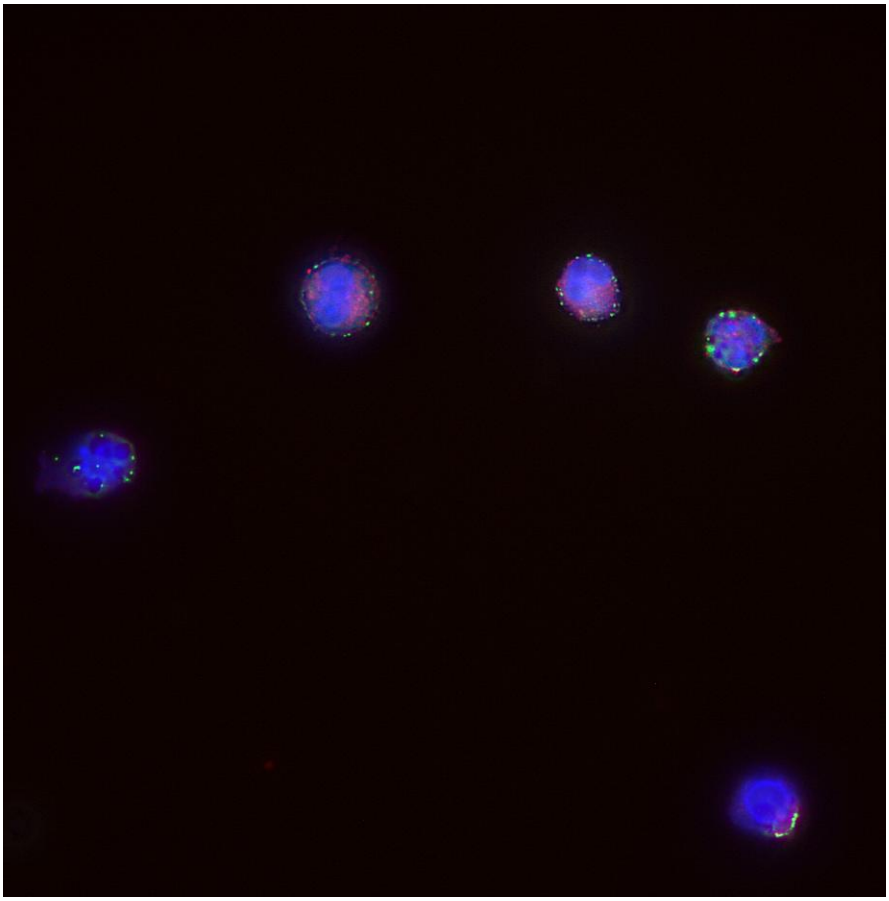

JAC + CD18 (ITGB2)
ERU

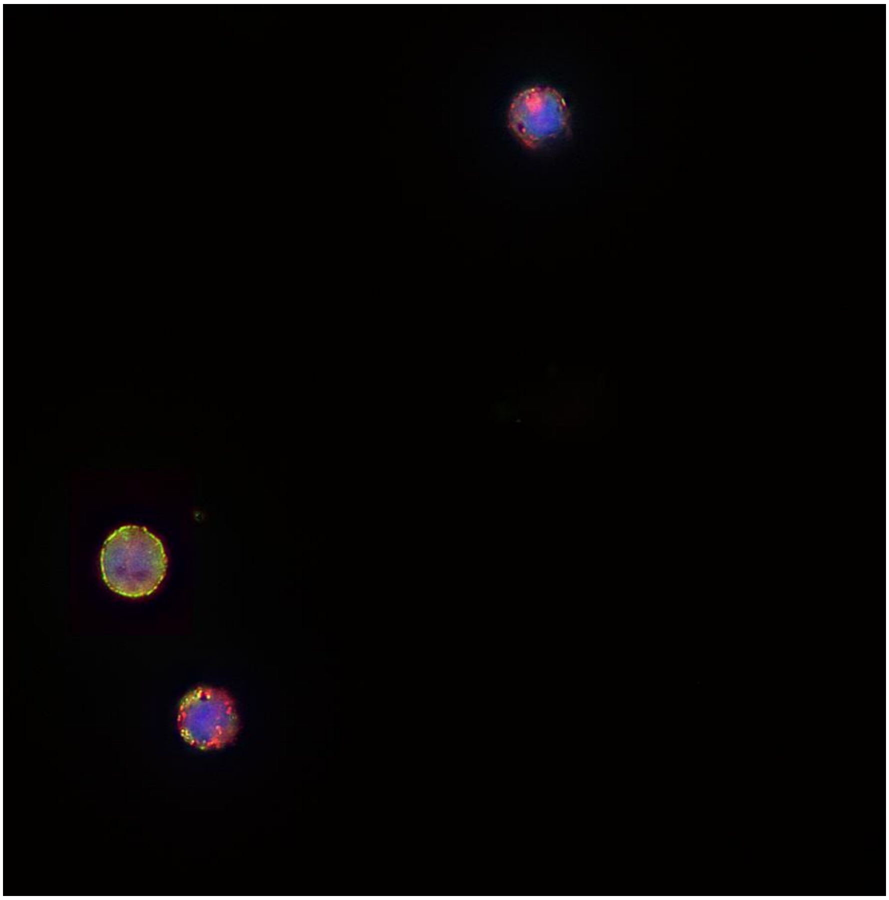

Supplement: Supplementary file 1 [file biomolecules-15-01444-s001.zip › RawData ICC.pdf]
