# Supplementary material for: Glycan Signatures on Neutrophils in an Equine Model for Autoimmune Uveitis
Source: Biomolecules. 2025 Oct 12;15(10):1444. doi: 10.3390/biom15101444 (PMC12562876; doi:10.3390/biom15101444)

Raw data of Western Blot analysis shown in Figure 5. Left: Eluates from JAC pull-down. Right: Supernatants from JAC pull-down and Input used for JAC pull-down.

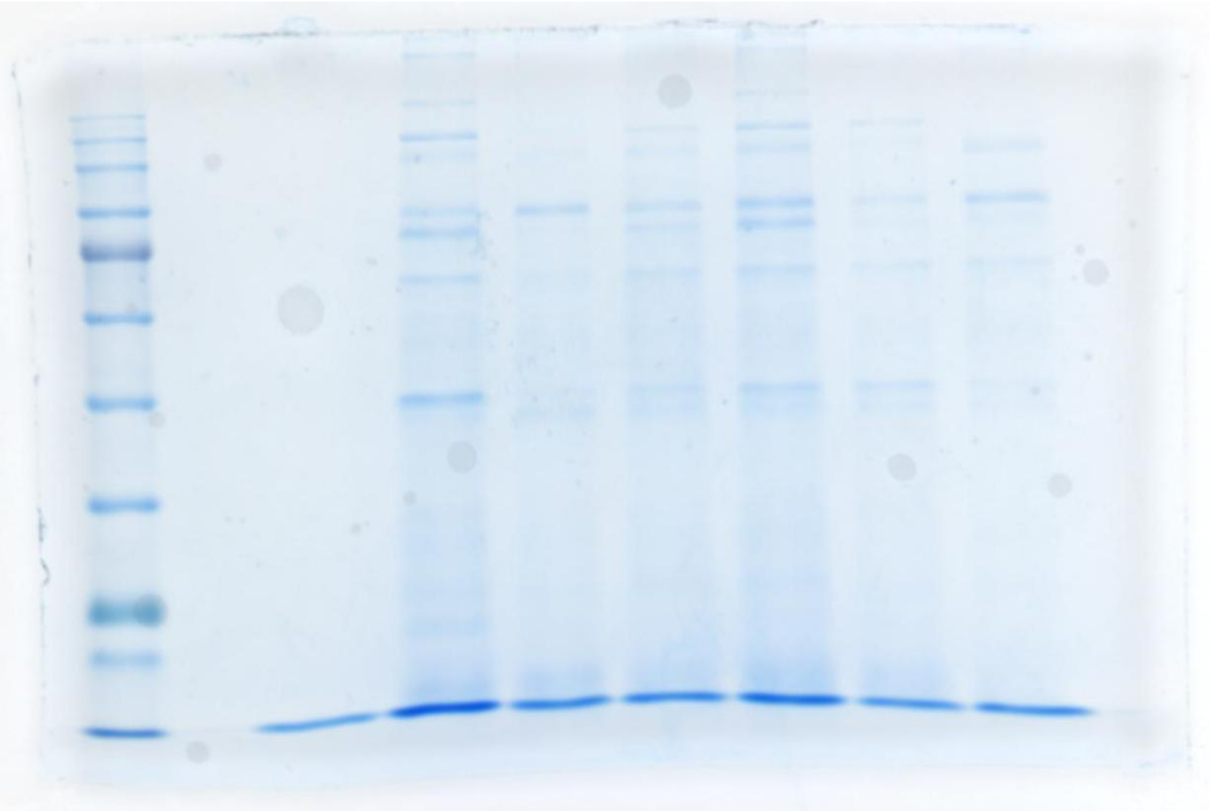

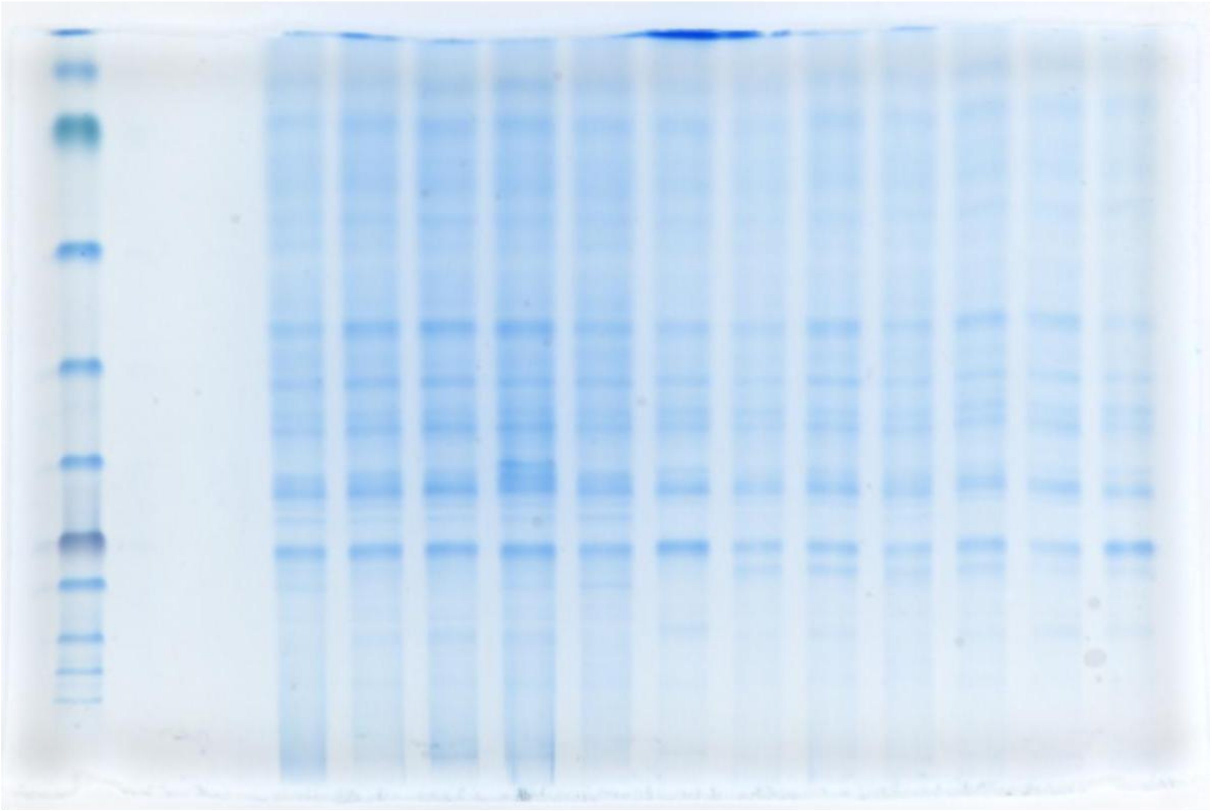

Supplement: Supplementary file 1 [file biomolecules-15-01444-s001.zip › RawData WB.pdf]
