# Supplementary material for: Glycan Signatures on Neutrophils in an Equine Model for Autoimmune Uveitis
Source: Biomolecules. 2025 Oct 12;15(10):1444. doi: 10.3390/biom15101444 (PMC12562876; doi:10.3390/biom15101444)
Supplement: Supplementary file 1 [file biomolecules-15-01444-s001.zip › Suppl Figures.pdf]

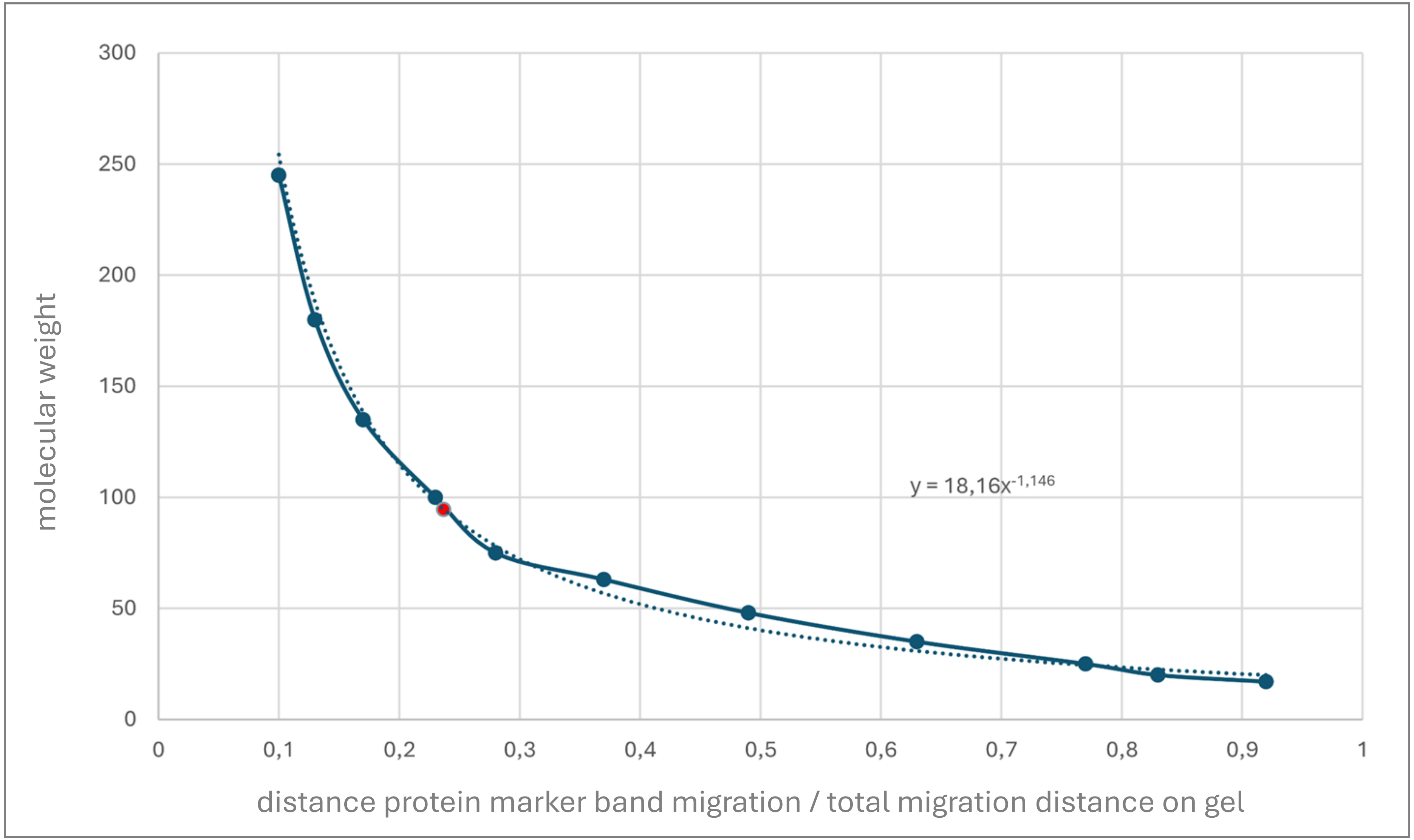

Figure S1: Calculation of the exact molecular weight of protein bands of interest was determined by generating a graph based on the migration distance of the protein marker bands (blue dots; distance protein marker band migration divided by total migration distance on gel) and subsequently allocating the migration distance of protein bands of interest on the graph (red dot at 94.5 kDa).

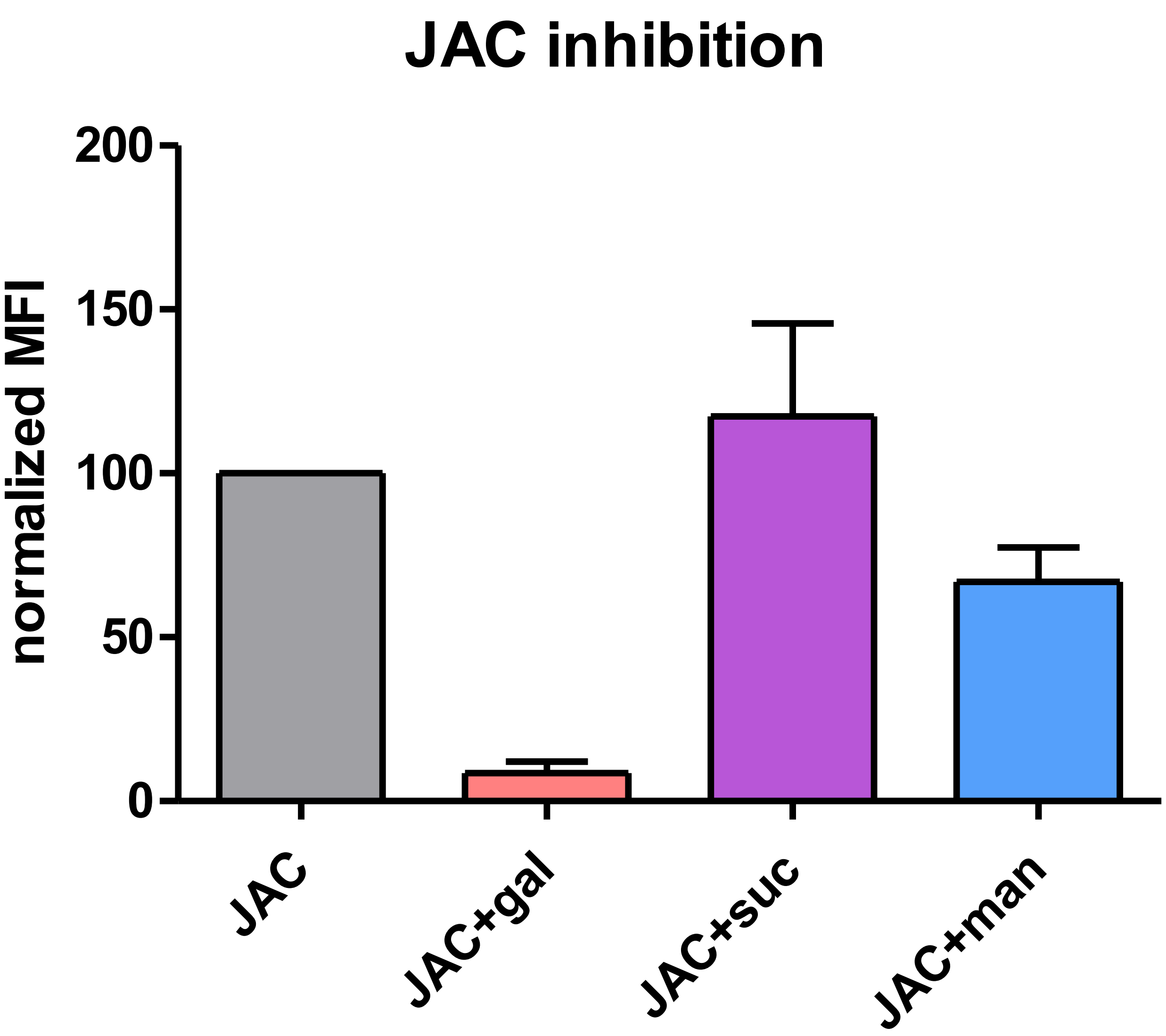

Figure S2: Bar graph showing JAC binding intensity to equine neutrophils measured by flow cytometry and shown as normalized mean fluorescence intensity (MFI). Binding of non-inhibited JAC is shown in grey. Strong decrease of JAC binding to equine neutrophils was observed after JAC-inhibition with galactose (gal; red bar). Inhibition with mannose (man; blue bar) showed slight decrease of JAC binding whereas sucrose (suc; violet bar) did not affect bind of JAC to the equine neutrophil surface. Binding intensity of gal, suc and man pre-treated JAC was normalized to non pre-treated JAC binding per animal (n=7; data was obtained in five independent experiments).
